# Dual-polarized ku-band microstrip antenna array with metamaterial loading and protective superstrate for GB-SAR applications

**DOI:** 10.1038/s41598-026-49752-0

**Published:** 2026-05-09

**Authors:** A. F. Desouky, A. S. Abd El-Hameed, A. R. Eldamak, T. G. Abouelnaga, H. M. Elhenawy

**Affiliations:** 1 Faculty of Engineering, Ain Shams University, Cairo, 11535 Egypt; 2 T. A. at Higher Institute of Engineering and Technology in Kafr Elshiekh (HIET), Kafr Elshiekh, 33612 Egypt; 3 Electronics Research Institute, El Nozha, Cairo Governorate 12662 Egypt

**Keywords:** GB-SAR, Dual polarization, Metamaterial, Patch antenna, SIW, SLL, ECC, Mutual coupling, Engineering, Materials science, Physics

## Abstract

This paper presents a high-performance 4 × 1 dual-polarized square-ring patch antenna array operating in the Ku-band for Ground-Based Synthetic Aperture Radar (GB-SAR) applications, with a compact size of 90 mm × 30 mm. The proposed design achieves compact integration by implementing dual-port polarization on a single substrate, enabling significant size reduction while effectively controlling mutual coupling and suppressing surface-wave propagation. To further enhance isolation and radiation performance, metamaterial unit cells (MTMLs) are introduced between adjacent elements to mitigate surface waves, while strategically positioned metallic vias improve port isolation. The antenna exhibits a wide impedance bandwidth from 16.3 to 18.5 GHz; however, the performance is specifically optimized within the 16.8–17 GHz band of interest for GB-SAR applications, where it achieves mutual coupling below − 12.5 dB, radiation efficiency exceeding 93%, and ECC values below 0.006. A two-layers dielectric superstrate are incorporated to improve radiation characteristics, resulting in a peak gain of 12 dBi, sidelobe suppression of − 16.8 dB, and a half-power beamwidth of 38.3°. These additional layers enhance gain and beam focusing while also serving as a protective shield against harsh automotive environmental conditions such as temperature variations, vibration, moisture, and mechanical stress. This protective layer ensures mechanical durability and stable RF performance without compromising radiation efficiency. The antenna array is fabricated and experimentally validated, with measured results showing strong agreement with simulations. The proposed configuration provides a compact, low-loss, high-efficiency, and environmentally effective solution suitable for advanced Ku-band radar applications.

## Introduction

Radar is a critical tool that supports the safety and progress of our modern world^1–4^. Ground-Based Synthetic Aperture Radar is a powerful remote sensing technology renowned for its high-precision, continuous monitoring of displacements in slopes, infrastructure, and glaciers. A standard GB-SAR system operates by moving two separate horn antennas along a linear rail to synthesize a large aperture for high-resolution imaging^5^. However, this configuration presents several practical challenges. The use of two horns, along with their associated hardware, directly contributes to a system that is bulky, heavy, and complex to transport and set up in the field^6–10^. Furthermore, most commercial systems utilize linearly polarized antennas due to their simplicity and lower cost. A key limitation of this linear polarization is its susceptibility to signal degradation and its low performance^11^. Despite these hardware limitations, the rapid data acquisition capability of GB-SAR remains vital for monitoring dynamic phenomena, such as changing atmospheric conditions and measuring vibrations in large structures like bridges and buildings.

The antenna is a critical component in the design of any radar system. Its fundamental role in radiating and capturing electromagnetic energy means its characteristics directly define key radar performance metrics, including gain, resolution, coverage, and beam shape^12,13^. Consequently, the selection of an appropriate antenna type is a primary design decision. Radar technology employs a diverse range of antenna architectures to meet different operational needs. Common types include the parabolic reflector antenna, which features high gain and narrow beamwidth, making it ideal for long-range detection. Another key type is the slotted waveguide antenna, valued for its low-profile, high-power handling, and robustness—characteristics essential for airborne and military applications^10,14^. Furthermore, phased array antennas are increasingly dominant in modern systems for their ability to electronically steer the beam with exceptional speed and agility, without any moving parts. Each of these designs offers a unique set of trade-offs, making the antenna not just a part of the system, but a determinant of its overall capability and application^15–18^.

Microstrip antennas have emerged as a compelling alternative for GB-SAR systems. Their primary advantages include a very low profile, light weight, and a planar structure that is highly amenable to integration, allowing for a more portable and rapidly deployable system. Furthermore, microstrip technology facilitates the creation of low-cost, conformal arrays, enabling electronic beam steering without the need for a large, heavy linear rail^19,20^. A significant body of research has explored the integration of planar antennas, particularly microstrip patches, into GB-SAR systems to mitigate the bulk and weight associated with traditional horn and reflector antennas. This focus has successfully advanced the development of more compact and portable systems. However, a notable limitation persists across much of this literature: the investigated planar antennas are predominantly designed for linear polarization^21–25^.

Dual polarization in radar systems is becoming increasingly essential due to its ability to significantly improve performance in diverse applications, from weather monitoring to military surveillance. Enabling the radar to transmit and receive signals in two orthogonal polarization states enhances the radar’s ability to discriminate between different types of targets and environmental conditions^26–33^. This capability is particularly valuable in differentiating between weather phenomena, such as rain, snow, and hail, and distinguishing between different types of objects, such as vehicles or foliage, based on their unique scattering properties^34^. Moreover, dual polarization increases the radar’s resistance to interference, improves detection accuracy, and enhances the overall resolution of the system^20,35^. This feature is especially crucial in complex scenarios where traditional single-polarized systems struggle to provide clear, reliable data^36–39^. For GB-SAR, this translates to a profound improvement in imaging capabilities. Dual-polarized data allows for a more detailed analysis of surface structure, material properties, and moisture content, moving beyond simple geometric shape detection^19^. Furthermore, it is the foundational step towards achieving fully polarimetric operation, which captures the complete scattering matrix and provides the highest fidelity information for classifying different terrain types and man-made materials. While a significant body of research has successfully integrated planar antennas into GB-SAR to solve issues of size and weight, a notable limitation persists, these designs are almost exclusively single polarized.

This paper presents a dual-polarized patch antenna designed for Ku-band radar applications at 17 GHz. A compact single-layer dual-polarized antenna is first developed and then extended to a four-element antenna array. One of the main challenges in such arrays is radiation pattern distortion caused by surface-wave propagation in the feeding network. To overcome this issue, metamaterial unit cells (MTMLs) are integrated between the antenna elements to suppress surface waves and improve radiation performance. A four-element array incorporating six MTMLs is designed and analyzed, demonstrating enhanced gain and stable radiation characteristics. The proposed design offers a lightweight, cost-effective, and high-performance solution, and its performance is validated using CST Microwave Studio.

### Antenna requirements

In GB-SAR, antenna specifications are strongly application-dependent rather than fixed, because the azimuth and elevation HPBW determine the illuminated scene, angular coverage, and measurement geometry. For instance, in structural/ground monitoring GB-SAR systems, antennas are often selected to provide sufficient elevation coverage while controlling azimuth footprint, and published GB-SAR antenna designs report highly different HPBW values (e.g., 10° elevation and 105° azimuth in a Ku-band MIMO GB-SAR antenna array). Similarly, standardization documents for ground-based radar sensors also show multiple antenna radiation-pattern types with different beamwidths depending on monitoring needs. Therefore, targeting an antenna with an elevation HPBW of approximately 38° and an azimuth HPBW of about 50° provides a practical compromise for GB-SAR applications, enabling adequate scene illumination and angular coverage while maintaining controlled footprint and measurement geometry for moderate-area monitoring.

### Antenna design

This section provides an overview of the design concept of the proposed antenna. The antenna is based on a dual-polarized square-ring patch (SRP) structure implemented on a Rogers Duroid 5880 substrate to achieve compact size and high radiation efficiency at Ku-band frequencies. To mitigate the mutual coupling between the two orthogonal ports and improve port isolation, metallic vias are incorporated in the antenna structure. In addition, metamaterial (MTML) unit cells are introduced to suppress surface-wave propagation and enhance radiation performance. After optimizing the single-element configuration, the design is extended to a linear array to further increase the antenna gain. Finally, a dielectric superstrate is employed to improve beam focusing and reduce sidelobe levels. This systematic design process from the initial element to the optimized array ensures a compact, efficient, and high-performance antenna structure suitable for radar applications.

The detailed design process, including the antenna geometry, single-element evolution, metamaterial unit-cell implementation, feeding network, 2 × 1 array development, 4 × 1 array configuration, and superstrate enhancement are described in the following sections.

### Design geometry

**Fig. 1:**
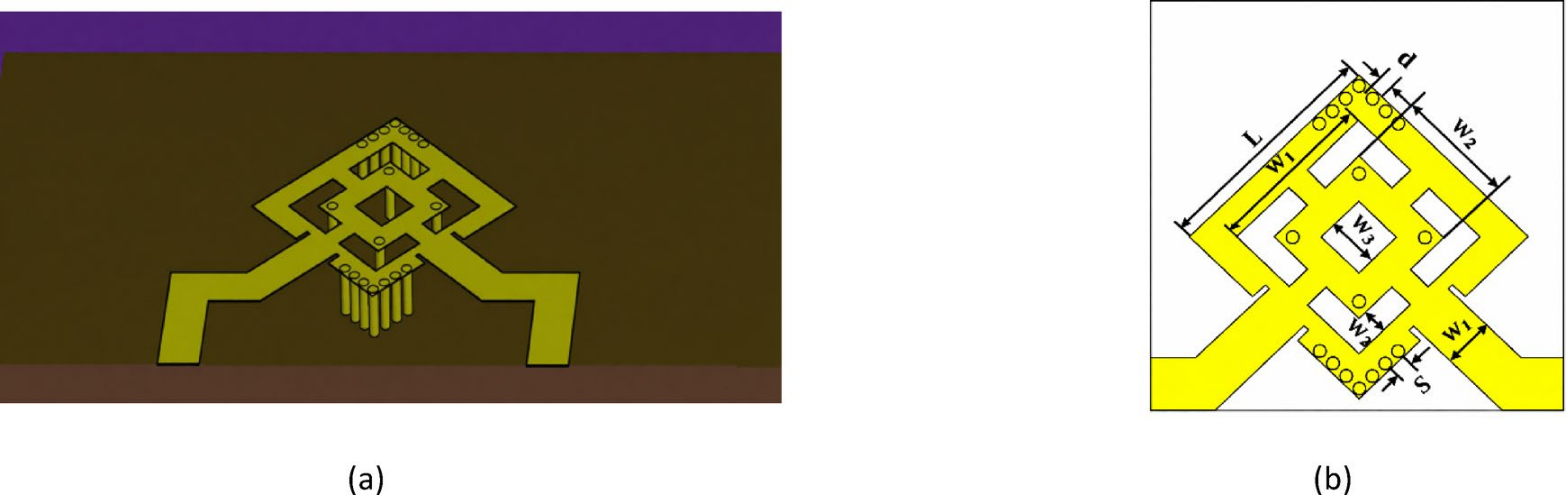
(**a**) 3D geometry of the proposed antenna structure and (**b**) Top view with parameters of the proposed antenna.

**Fig. 2:**
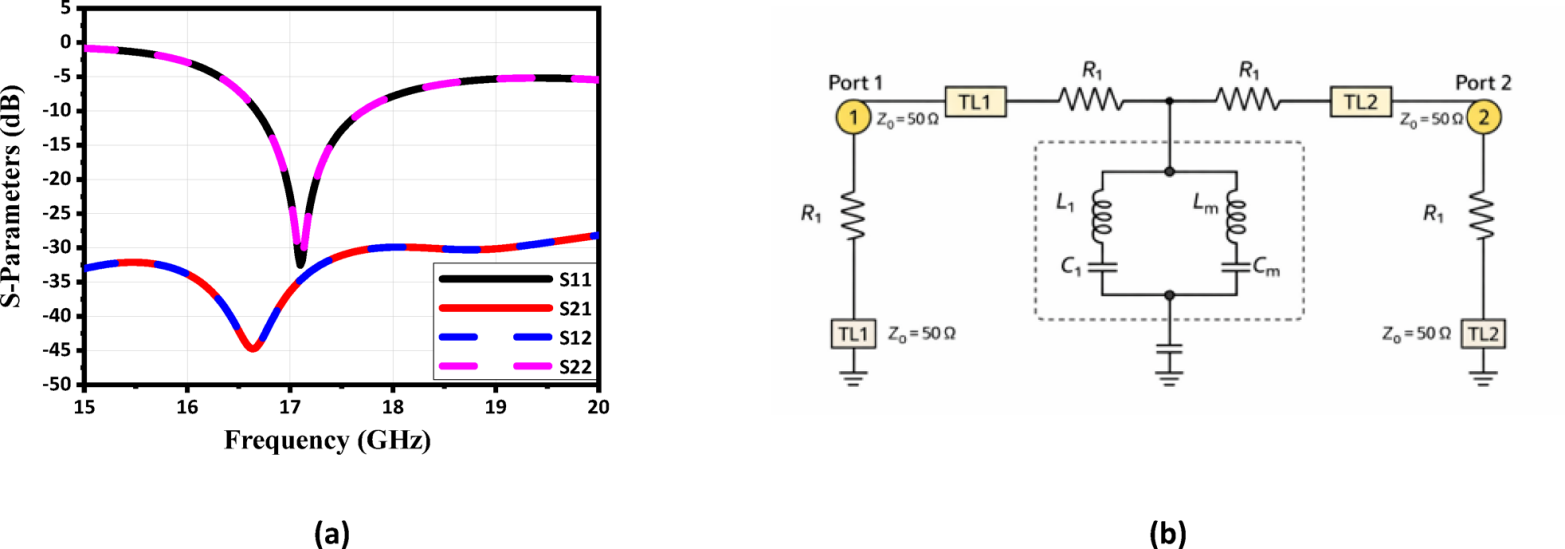
(**a**) The simulated reflection coefficient of the proposed antenna and (**b**) The equivalent circuit of the antenna design.

The proposed dual-polarized antenna is designed on RT Duroid 5880 substrate, which has a relative permittivity of 2.2 and a thickness of 1.57 mm. The antenna configuration is illustrated in Fig. 1. The structure incorporates a square-ring patch with nested sections to support dual polarization and mitigate surface-wave interference. A square patch antenna supports two orthogonal fundamental modes (TM₁₀ and TM₀₁), which have the same resonant frequency. The total size of the patch is defined by $\mathrm{L}=9 \mathrm{m}\mathrm{m}$, with inner square sections of dimensions ${\mathrm{W}}_{1}=5.78 \mathrm{m}\mathrm{m}$, ${\mathrm{W}}_{2}=3.78 \mathrm{m}\mathrm{m}$, ${\mathrm{W}}_{3}=2 \mathrm{m}\mathrm{m}$, and ${\mathrm{W} }_{4}=1\mathrm{m}\mathrm{m}$. The antenna is excited through two orthogonal feeding lines, each having a width of ${\mathrm{W}}_{\mathrm{f}}=2 \mathrm{m}\mathrm{m}$, ensuring impedance matching and dual-polarization functionality. The optimization aimed to achieve a balance between surface-wave suppression, port isolation, and impedance matching. Increasing the number of vias improves surface-wave confinement but may affect impedance and fabrication complexity; therefore, an optimal number was selected to maximize isolation while preserving radiation efficiency. Periodic vias are embedded around two opposite corners of the proposed SRP, with a spacing of $\mathrm{S} =0.7 \mathrm{m}\mathrm{m}$ and a via diameter of $\mathrm{d} =0.5 \mathrm{m}\mathrm{m}$, and total number of vias were determined through a comprehensive parametric optimization process.The diagonal placement was chosen based on surface current distribution analysis, where each via is positioned in a high-current region for one polarization mode and simultaneously near a low-current (nodal) region for the orthogonal mode. This configuration allows the vias to act as short-circuit boundaries for suppressing unwanted surface waves while minimally disturbing the orthogonal polarization mode, thereby enhancing isolation and radiation stability. The antenna simulated response is presented in Fig. 2a. The reflection coefficient remains below −10 dB throughout the operating band from 16.9 to 17.8 GHz, with a resonance occurring at approximately 17 GHz. In addition, the isolation stays above 30 dB across the entire frequency range. The proposed dual-fed patch antenna can be represented by a two-port parallel RLC resonator model, as illustrated in Fig. 2b. Each feed line is modeled as a 50-Ω transmission line exciting a common radiating resonator characterized by radiation resistance ${\mathrm{R}}_{1}$, inductance ${\mathrm{L}}_{1}$, and capacitance ${\mathrm{C}}_{1}$, corresponding to the dominant TM_10_ mode of the patch. The etched slots and structural perturbations modify the current distribution and introduce additional reactive loading, which is incorporated into the equivalent inductive and capacitive elements. The electromagnetic interaction between the two ports is represented by mutual inductance ${\mathrm{L}}_{\mathrm{m}}$ and mutual capacitance ${\mathrm{C}}_{\mathrm{m}}$, accounting for magnetic and electric coupling mechanisms, respectively. These coupling elements determine the port isolation and influence the transmission coefficient ${\mathrm{S}}_{21}$. Reduced values of ${\mathrm{L}}_{\mathrm{m}}$and ${\mathrm{C}}_{\mathrm{m}}$correspond to improved isolation performance. This circuit model provides physical insight into the antenna behavior, impedance matching characteristics, and mutual coupling suppression of the proposed antenna structure.

### Single element design procedure

**Fig. 3:**
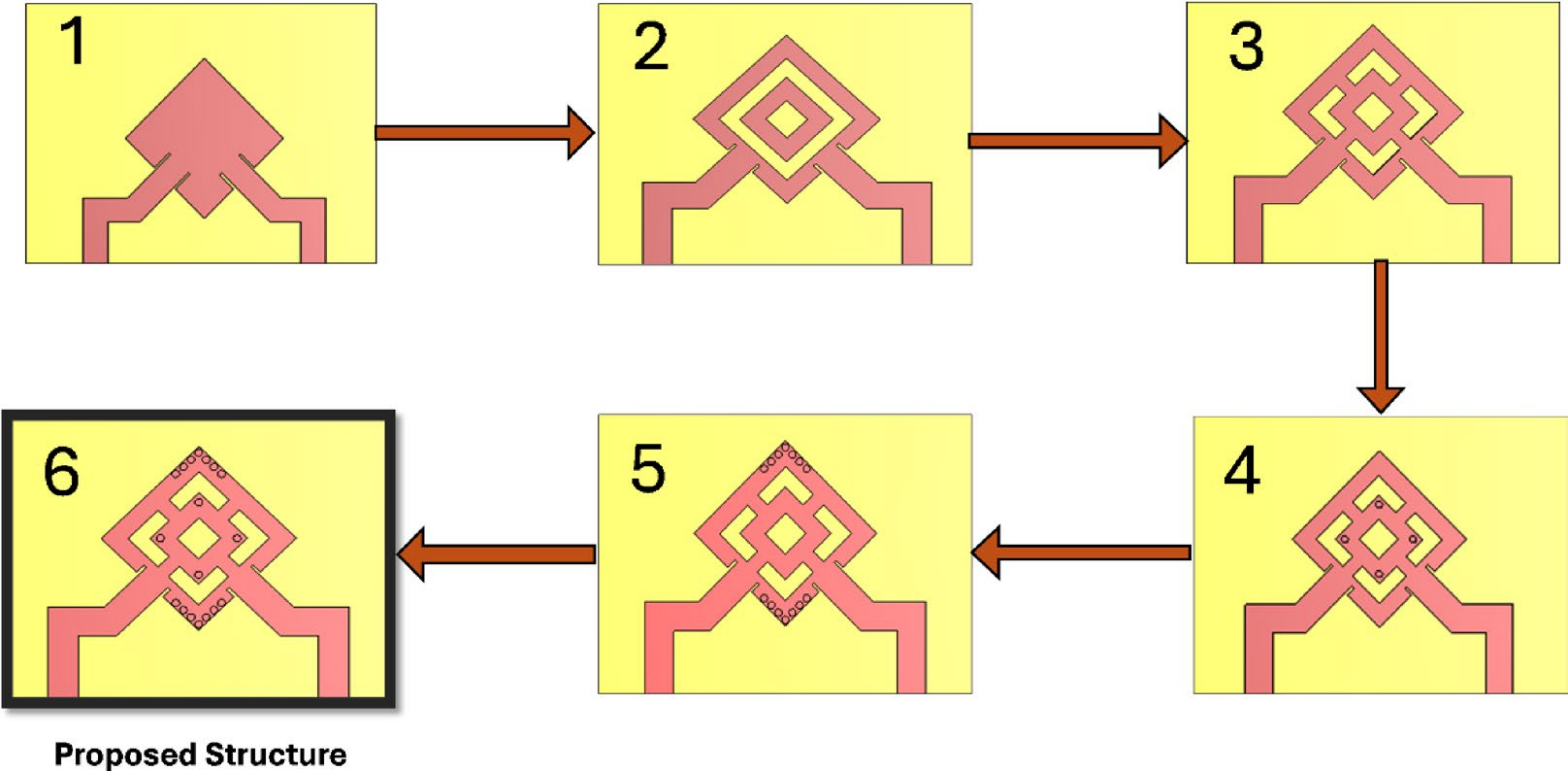
Design procedures of the single element.

**Fig. 4:**
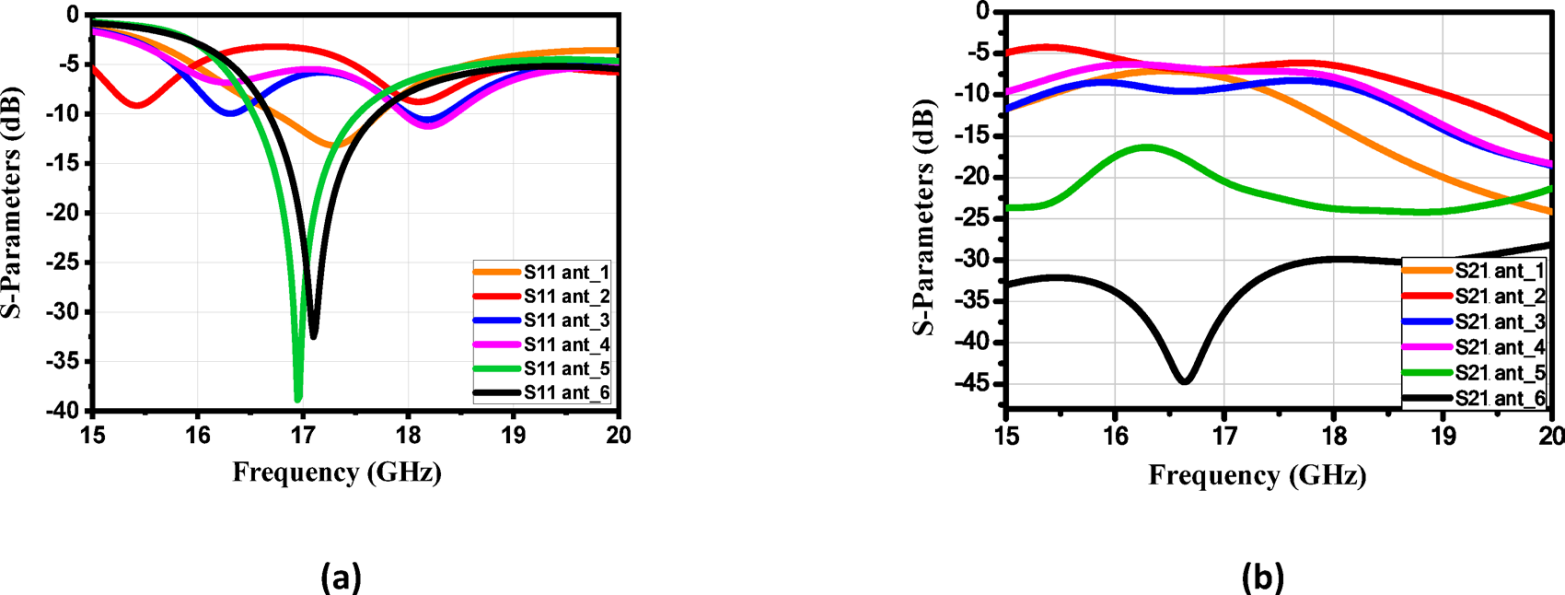
(**a**) The simulated reflection coefficient of the six stages of theproposed antenna and (**b**) The transmitted reflection coefficient of the sixstages of the proposed antenna.

**Fig. 5:**
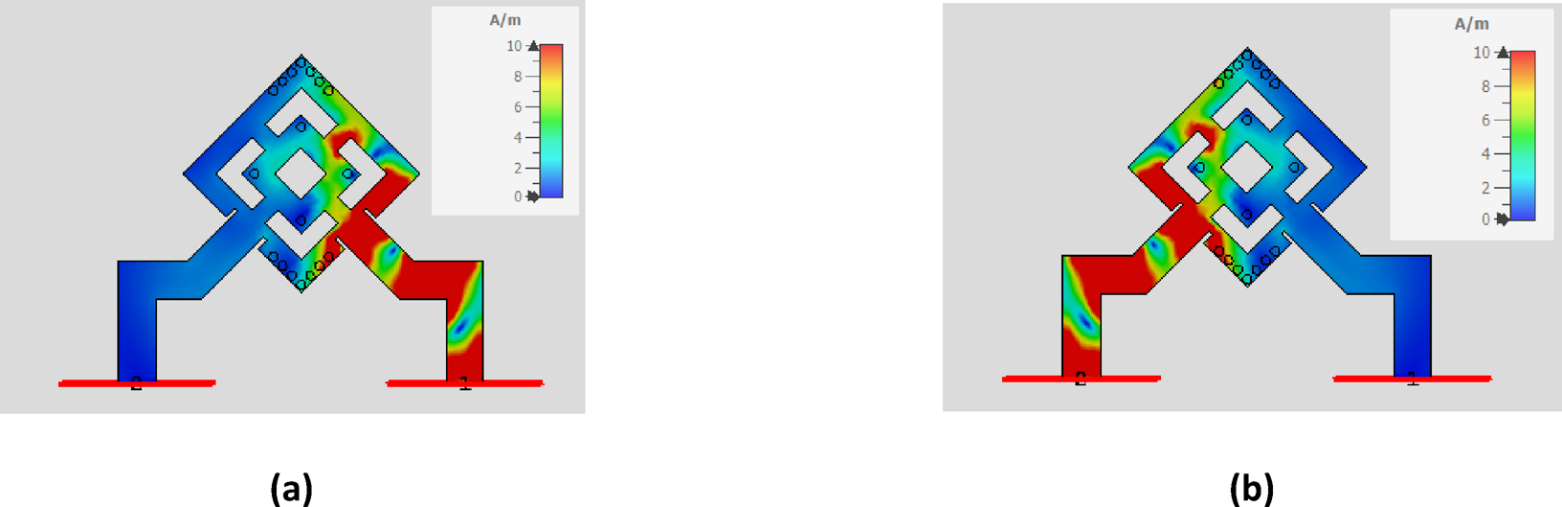
Surface current distribution on the proposed single element at 17 GHz (**a**) Port 1 (**b**) Port 2.

**Fig. 6:**
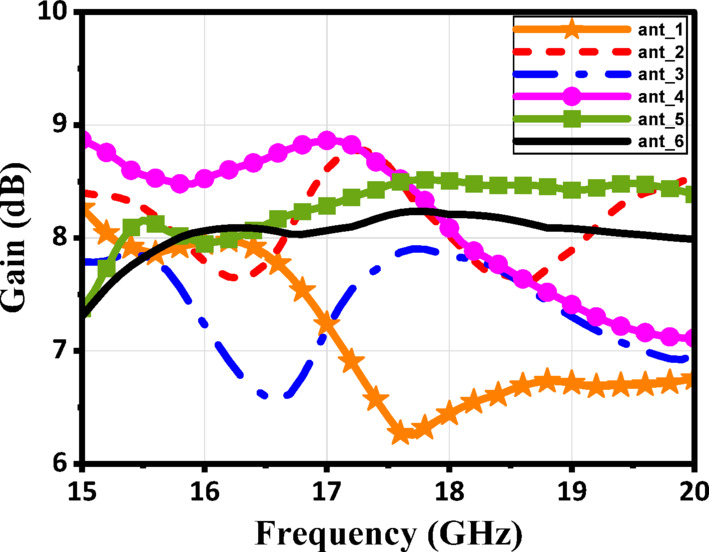
The simulated gain response of the six stages of the proposed antenna.

Extensive simulations were conducted as part of the initial analysis to evaluate the impact of various design changes on the antenna’s performance. The design procedures are illustrated in Fig. 3. The S-parameters of the six stages of the proposed structures are shown in Fig. 4a and b, respectively. The antenna design procedures were carried out in six sequential stages to enhance performance. In the initial stage, the rotated square patch exhibited weak resonance and poor impedance matching, with high coupling between ports. Subsequent modifications in stages 2 and 3 improved the S11 response near 17 GHz and slightly reduced coupling, though matching remained shallow and bandwidth narrow. Further refinements in stages 4 and 5 strengthened the resonance, achieving deeper S11 values (around − 20 dB) and noticeably better port isolation. Finally, the proposed structure in stage 6 (ant_6) demonstrated significant improvement, with excellent impedance matching (S11 ≈ − 35 dB at 17 GHz) and strong port isolation (S21 < − 40 dB). These results confirm that the introduced antenna, minimized coupling, and optimized the antenna for dual-polarized operation. Figure 5 illustrates the current distribution of the proposed single antenna element at 17 GHz when excited individually from Port 1 (a) and Port 2 (b). This limited cross-excitation indicates low mutual coupling between Port 1 and Port 2. Therefore, the structure provides good isolation between the two feeding ports. The construction of a linear antenna array necessitates a feeding network, which inherently excites surface waves. Mitigating these waves requires a different approach than the via-based method used for a single patch element. For this purpose, we have selected a metamaterial-based technique. Figure 6 presents the simulated gain evolution corresponding to the six design stages of the proposed antenna element. The initial configuration (Stage 1) provides a moderate gain of approximately 7.5–8.0 dB near 17 GHz, with noticeable fluctuation across the band. As the geometry is progressively modified (Stages 2–4), the gain improves and stabilizes around 8.0–8.8 dB due to enhanced current distribution and stronger resonance formation. Further refinement in Stage 5 results in improved radiation behavior, while the final optimized structure (Stage 6) achieves a stable gain of approximately 8.2–8.5 dB around the target frequency of 17 GHz. The progressive enhancement confirms the effectiveness of the adopted design methodology and demonstrates the contribution of each structural modification to overall radiation performance.

### Metamaterial (MTML) cell

**Fig. 7:**
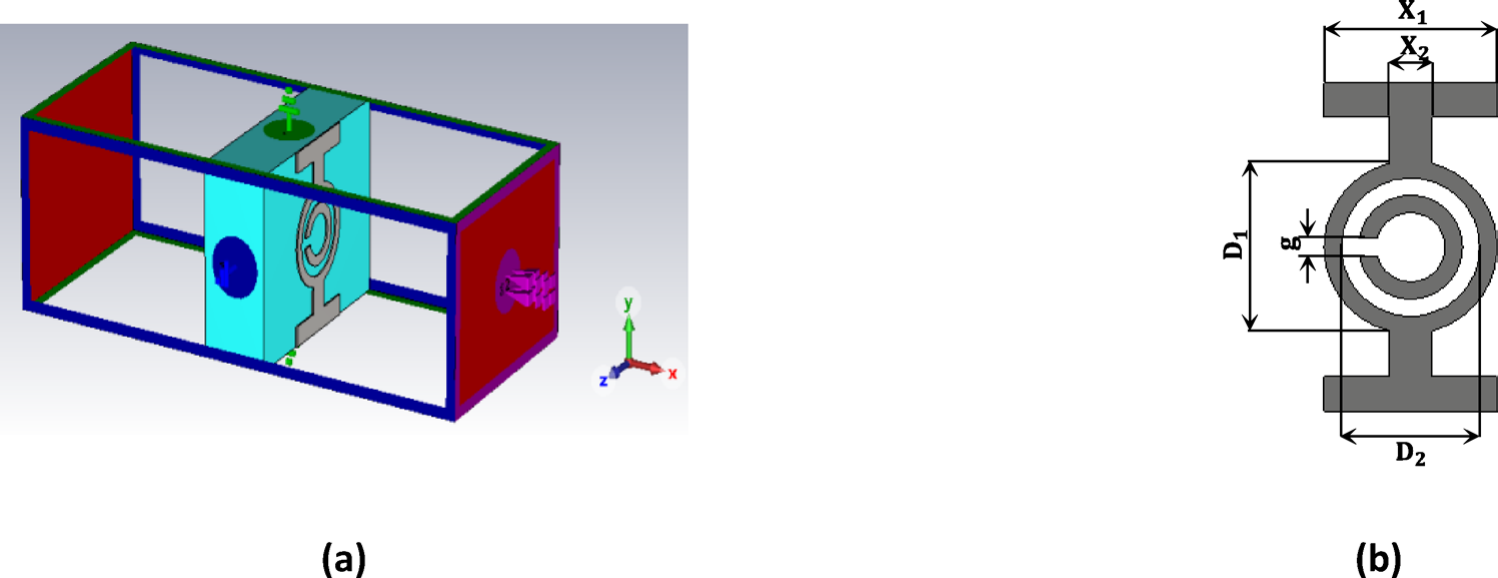
(**a**) The structure of the proposed MTML unit cell, (**b**) The design ofthe MTML structure specification.

**Fig. 8:**
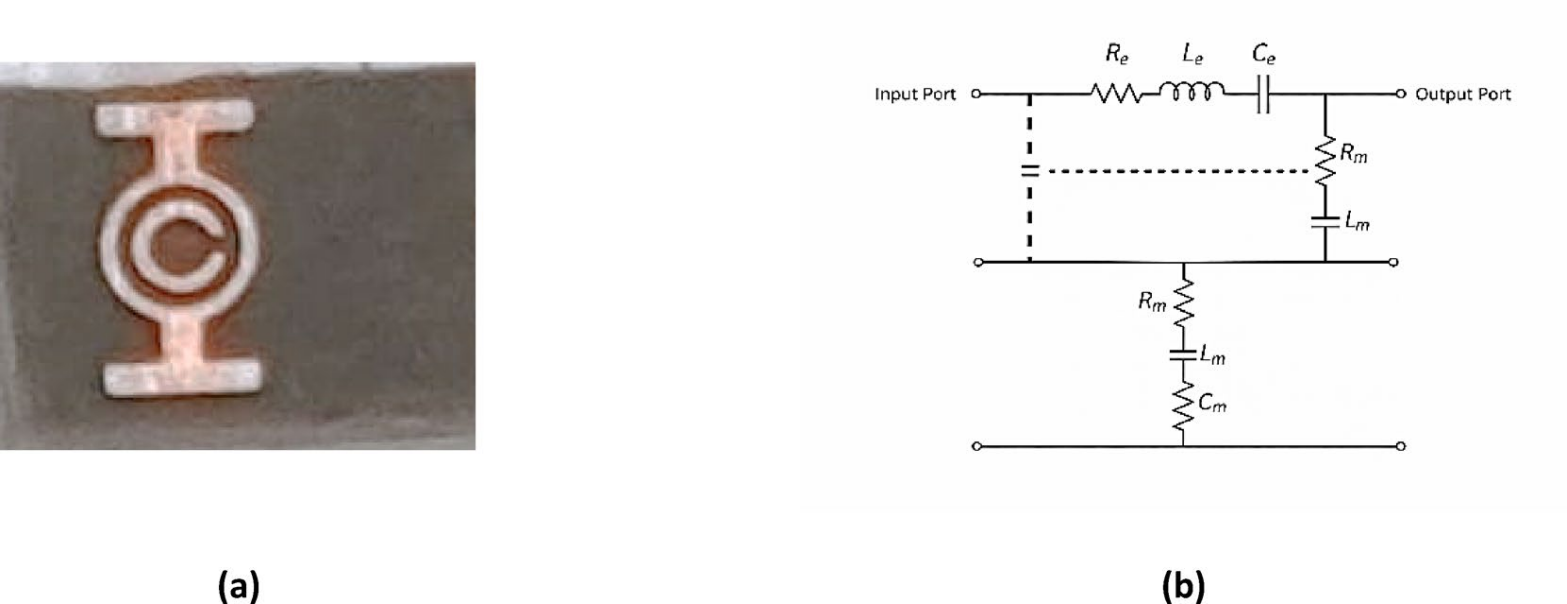
(**a**) The fabrication of the proposed MTML unit cell and (**b**) The RLC equivalent circuit of MTML unit cell.

The MTML cell comprises a capacitively loaded strip (CLS), a modified split-ring resonator (SRR), and a wire. Together, they enable the achievement of negative $\mathcal{E}$ and ${\upmu }$^40,41^. This configuration leads to a remarkable negative index of refraction, which increases the radiating power of the proposed antenna. The cell is designed using the cost-effective Duroid 5880 substrate material. The total dimensions of the structure are 5 mm × 3.8 mm × 1.57 mm. The MTML unit cell uses a split-ring resonator (SRR) configuration, which includes two concentric ring loops. The inner loop is smaller and features a gap at its center, which creates a capacitance that influences the resonant behavior of the metamaterial. Capacitive Loaded Strips (CLS) are I-shaped conductive strips that resemble elongated metallic wires. Combining the two structures allows dual resonance since the CLSs resonate with a parallel electric field, and the SRR reacts to a perpendicular magnetic field^42,43^. This paper will utilize a single band that aligns with the antenna’s operating frequency of 17 GHz. The MTML unit cell was simulated using CST Microwave Studio, and the simulation geometry is displayed in Fig. 7a. Along the x-axis, an electromagnetic wave was excited while the structure was positioned between two waveguide ports. Perfectly conducting boundary conditions were implemented on the walls perpendicular to the y-axis (electrical) and z-axis (magnetic). The impedance is normalized to 50 Ω. The parameters of the ring MTML are displayed in Fig. 7b, where ${\mathrm{X}}_{1}=2 \mathrm{m}\mathrm{m}$, ${\mathrm{X}}_{2}=0.5 \mathrm{m}\mathrm{m}$, ${\mathrm{D}}_{1}=2 \mathrm{m}\mathrm{m}$, ${\mathrm{D}}_{2}=1.6 \mathrm{m}\mathrm{m}$, and $\mathrm{g}=0.2 \mathrm{m}\mathrm{m}$. Figure 8a shows the MTML unit cell after fabrication. Figure 8b shows the Equivalent RLC circuit model of the proposed MTML unit cell. The parallel RLC branch $\left({\mathrm{R}}_{\mathrm{e}},{\mathrm{L}}_{\mathrm{e}},{\mathrm{C}}_{\mathrm{e}}\right)$represents the electric resonance of the capacitive loaded strips (CLS), while the series RLC branch $\left({\mathrm{R}}_{\mathrm{m}},{\mathrm{L}}_{\mathrm{m}},{\mathrm{C}}_{\mathrm{m}}\right)$models the magnetic resonance of the split-ring resonator (SRR).

**Fig. 9:**
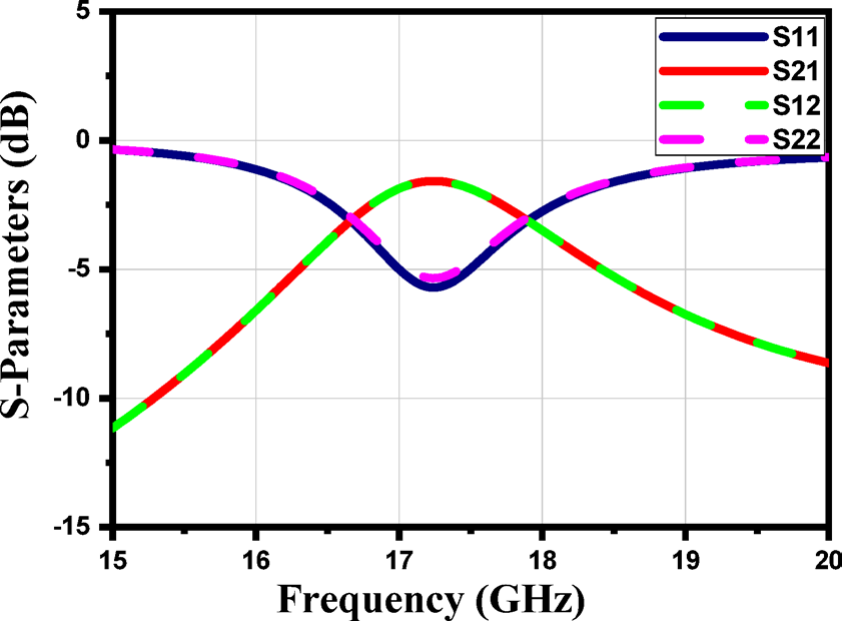
The magnitude of the S-parameters for the suggested MTML unitcell.

Figure 9 shows the S-parameters. The constitutive effective parameters, such as the relative permeability ${{\upmu }}_{\mathrm{r}}$, the relative permittivity${\mathcal{E}}_{\mathrm{r}}$, and their refractive index ${n}_{r}$, were extracted from S11 and S21 using the “Nicolson-Ross-Weir” approach^41–43^.

1 $${\mathcal{E}}_{r}=\frac{2}{j{k}_{o}d}*\frac{1-{V}_{1}}{1+{V}_{1}}$$

2 $${\mu }_{r}=\frac{2}{j{k}_{o}d}*\frac{1-{V}_{2}}{1+{V}_{2}}$$

3 $${n}_{r}=\sqrt{{\mathcal{E}}_{r}{ \mu }_{r}}$$

4 $${V}_{1}= {S}_{21}+{S}_{11}$$

5 $${V}_{2}= {S}_{21}-{S}_{11}$$

**Fig. 10:**
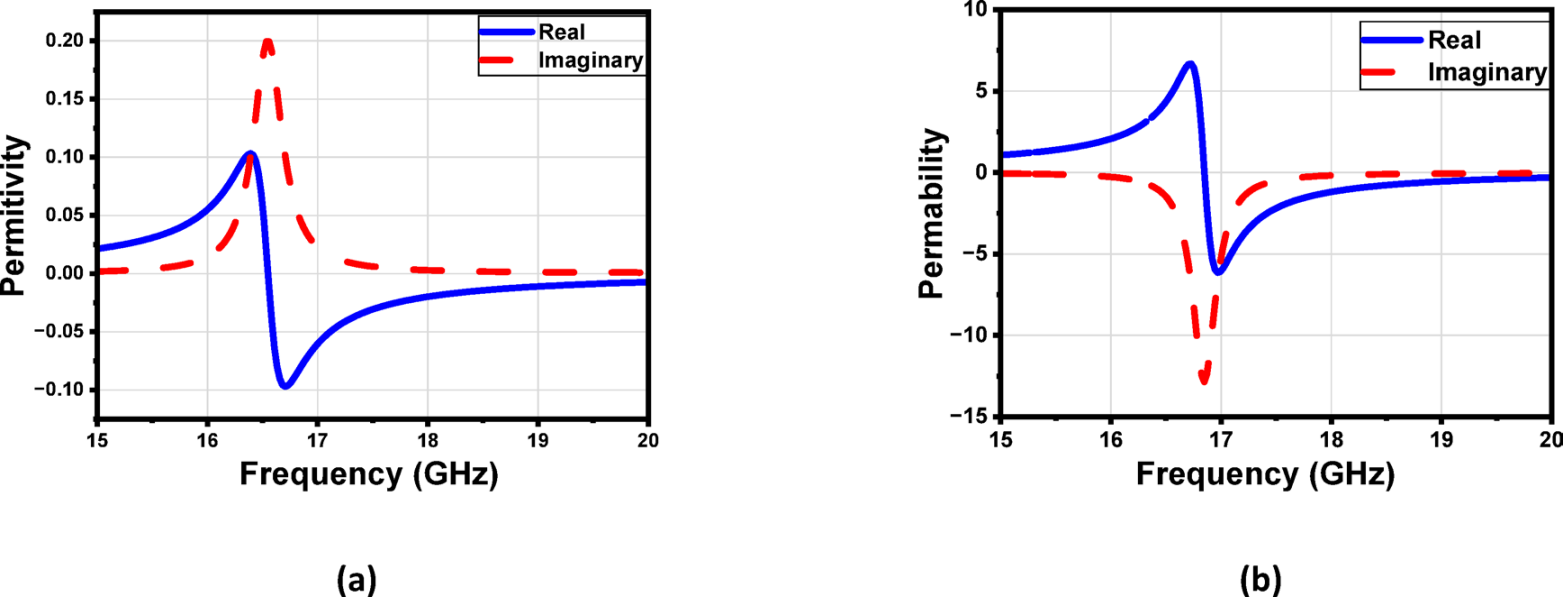
The proposed unit cell (**a**) permittivity ${\mathcal{E}}_{\mathbf{r}}$ and (**b**) permeability ${\boldsymbol{\upmu }}_{\mathbf{r}}$ .

Where ${\mathrm{k}}_{\mathrm{o}}=\omega ⁄C$, d represents the slab thickness and C is the speed of light. A Matlab script has been created to compute the relative permittivity ${\mathcal{E}}_{\mathrm{r}}$ and relative permeability ${{\upmu }}_{\mathrm{r}}$ from the S-parameters. The S-parameters in Fig. 9 show a clear resonance around 17 GHz, indicating that the unit cell exhibits strong electromagnetic interaction at this frequency. In Fig. 10, the extracted effective permittivity and permeability both show negative real values near the same resonance point, confirming the presence of electric and magnetic resonances. Together, these results demonstrate that the proposed structure behaves as a MTML unit cell with distinct MTML characteristics around 17 GHz.

### Feeding network

**Fig. 11:**
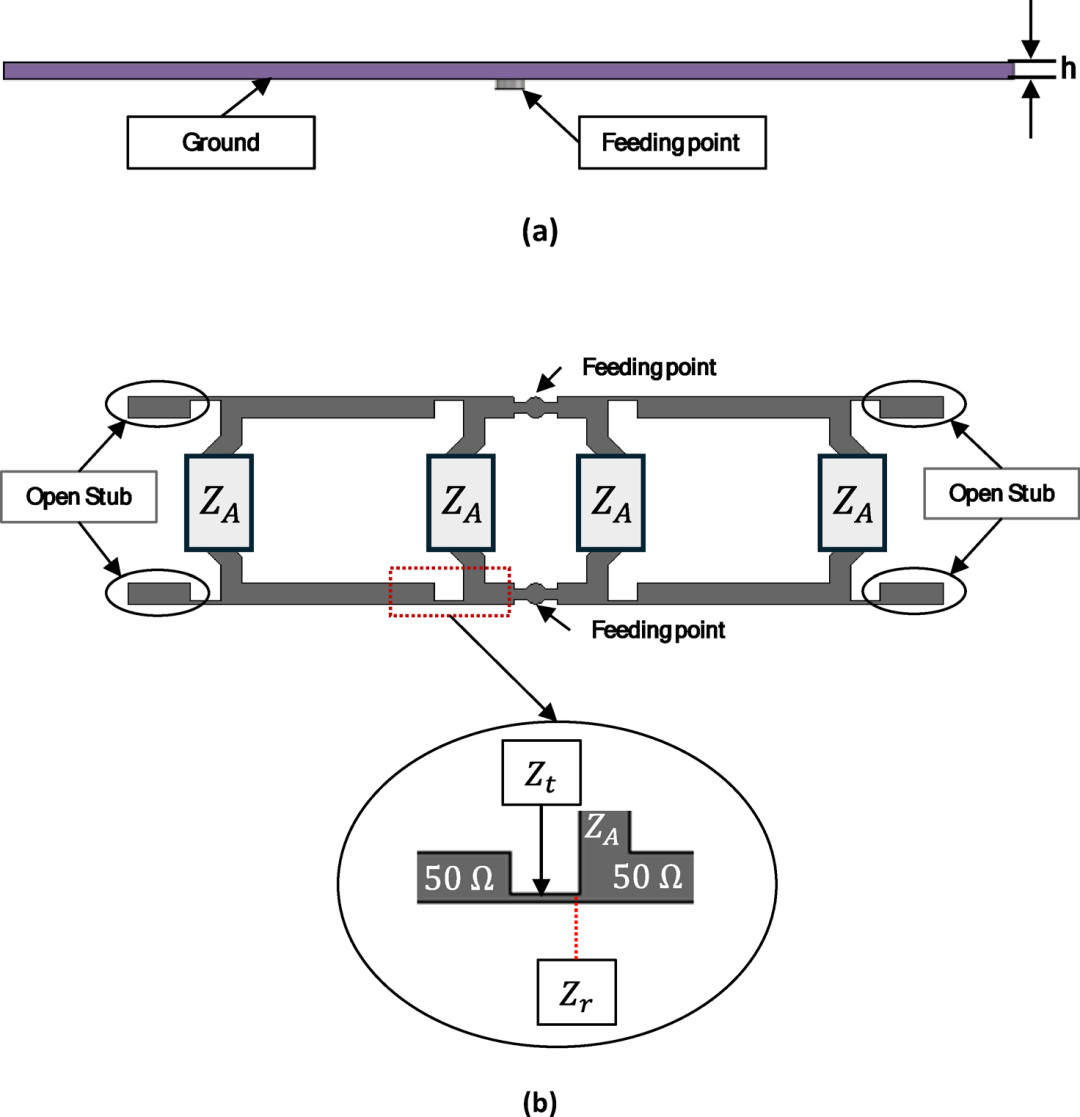
Antenna array geometry. (**a**) Side view (**b**) Feeding network.

The structure of a linear antenna array centrally excited by a traveling wave-feeding network is shown in Fig. 11. The geometry of the 4 × 1 antenna array and its feeding network was derived analytically using transmission-line theory and array factor principles before full-wave optimization. The inter-element spacing was selected as approximately ${\lambda }_{g}$at 17 GHz to ensure constructive broadside radiation while suppressing grating lobes and minimizing sidelobe levels (SLL).

The array is excited using a traveling-wave feeding structure, where power is progressively distributed along the main transmission line. Unlike a resonant array, which introduces many possibilities for deterioration of the radiation pattern, the traveling wave-feeding structure offers a wide bandwidth and high efficiency, which is especially useful for remote sensing applications.

The impedance of each radiating element was first designed to be 50 Ω at the operating frequency. A quarter-wave impedance transformer was then introduced to match the 50 Ω feed line to the patch input impedance. The transformer impedance was calculated using:

6 $${Z}_{t}=\sqrt{{Z}_{0}{Z}_{A}}$$

where ${Z}_{0}=50{\hspace{0.17em}}{\Omega }$ is the characteristic impedance of the main feed line, and ${Z}_{A}$is the input impedance of the antenna element. To achieve amplitude tapering and sidelobe suppression, two λg/2 open-circuit stubs are added to the array to reflect the power traveling toward the ends. The amplitude of each radiating element is governed by:

7 $$\left|Ai\right| ={ k}^{(i-1)} + {k}^{(2N-i)}, i = 1,\ldots ,N$$

8 $$k = 1 -\left(\frac{Zo }{ {Z}_{A}}\right)$$

After establishing these analytical dimensions, the geometry was fine-tuned through electromagnetic simulation to account for mutual coupling and fabrication tolerances.

The key parameter in this model is the coupling coefficient, k which dictates the proportion of power distributed to each radiating element. Its value is computed using Eq. (8), where ${Z}_{o}$ represents the 50 Ω characteristic impedance of the primary feed line, and ${\mathrm{Z}}_{\mathrm{A}}$ is the input impedance of the patch antenna at its feed point, illustrated in Fig. 11a. The second term in Eq. (7) specifically accounts for the power reflected from the λg/2 open-circuit stub; this reflected power constructively interferes at the feed point as it returns in phase. The value of the coupling coefficient $k$ was initially derived analytically and subsequently optimized through parametric simulation using CST software, where $k=5/6$ was found to provide the best compromise between sidelobe suppression and radiation efficiency. The primary radiating component of the antenna array is the square patch antenna. The optimal k value is satisfied by choosing an impedance of ${\mathrm{Z}}_{\mathrm{A}}=300{\Omega }$. The patch’s impedance, which is parallel to the impedance at the reference ${\mathrm{Z}}_{\mathrm{r}}=60{\Omega }$, precisely matches the border elements’ characteristic impedance and helps to reduce SLL. As seen in Fig. 11b, an impedance of 60 Ω can be obtained by matching the feeding line impedance of 50 Ω to the quarter-wave transformer impedance ${\mathrm{Z}}_{\mathrm{t}}=54.8{\Omega },$. Each radiating element is spaced λg apart.

### 2 × 1 Array development

#### Two-element antenna array without MTML

**Fig. 12:**
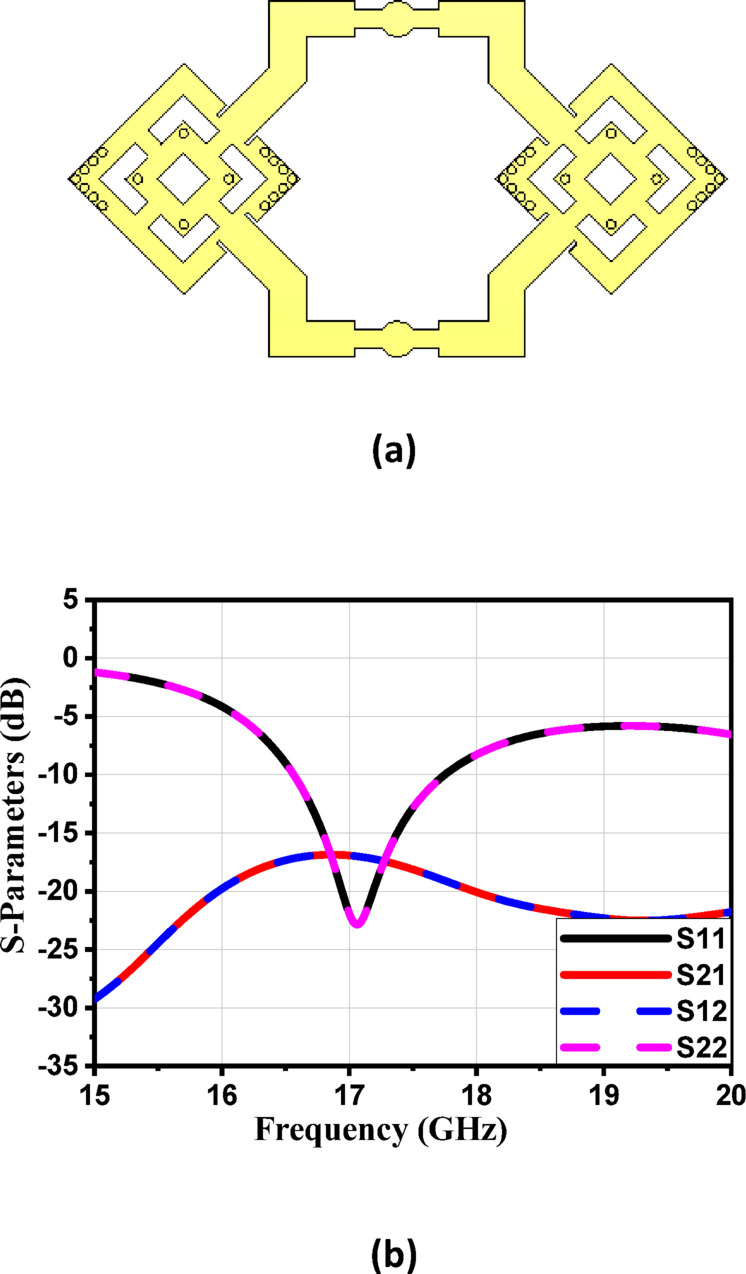
(**a**) The 2 × 1 antenna array proposed structure and (**b**) Simulated S-parameters of the proposed array.

**Fig. 13:**
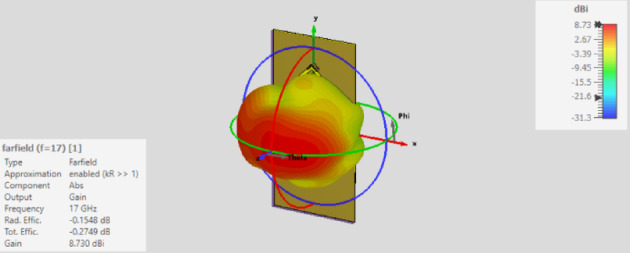
The maximum gain at 17 GHz of the proposed antenna array.

The suggested dual-polarized antenna element is used in the design of a 2 × 1 antenna array to improve overall performance. The array operates in the frequency range of 16.5 –7.8 GHz, with a resonance frequency of 17.1 GHz. The structural layout of the 2 × 1 antenna array is shown in Fig. 12a, while Fig. 12b presents the simulated reflection coefficients, demonstrating effective impedance matching across the operating band. The array configuration employs a traveling wave-feeding network, ensuring wideband operation and improved port isolation. However, mutual coupling between the antenna elements remains challenging, affecting radiation pattern stability and overall performance. Additionally, surface waves generated within the feeding network contribute to radiation pattern distortion, further impacting the array’s efficiency. The radiation pattern of the proposed antenna array is uneven, and its gain is relatively low, peaking at 8.8 dBi as shown in Fig. 13. To address these issues, a metamaterial-based approach is introduced to effectively reduce mutual coupling and suppress surface waves, improving radiation characteristics and overall antenna performance.

### Two-element antenna array loaded with MTML

**Fig. 14:**
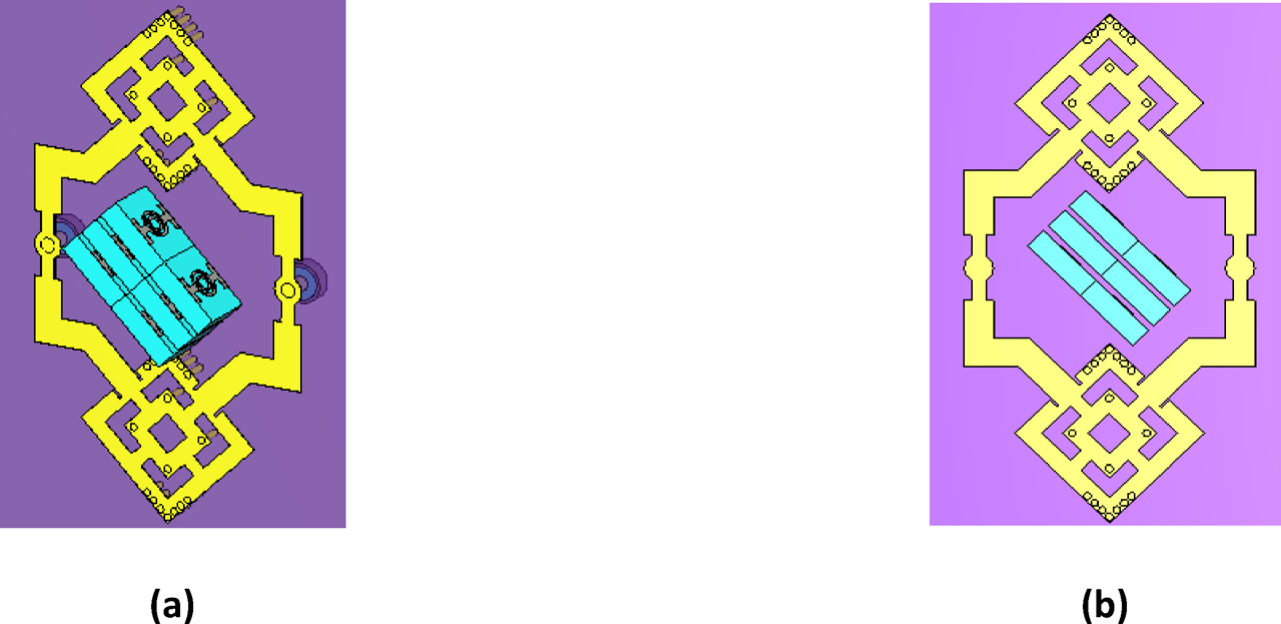
The proposed antenna array with proposed MTML unit cells.

**Fig. 15:**
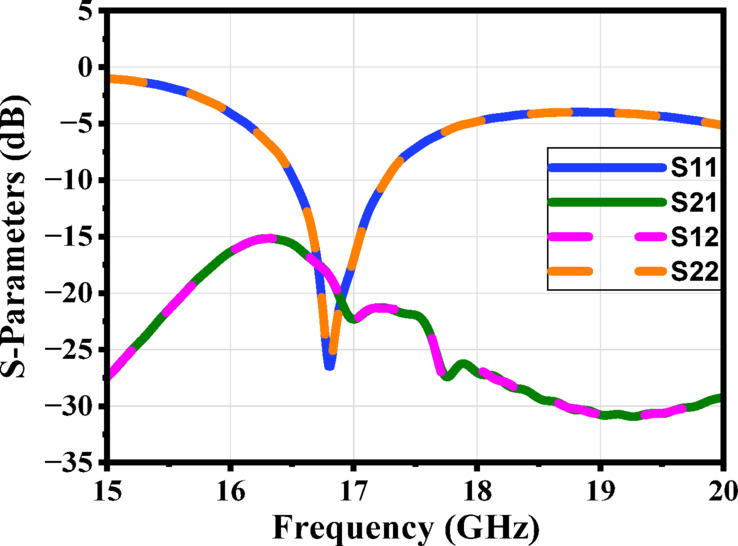
The simulated S-parameters for the antenna array design incorporating MTML.

**Fig. 16:**
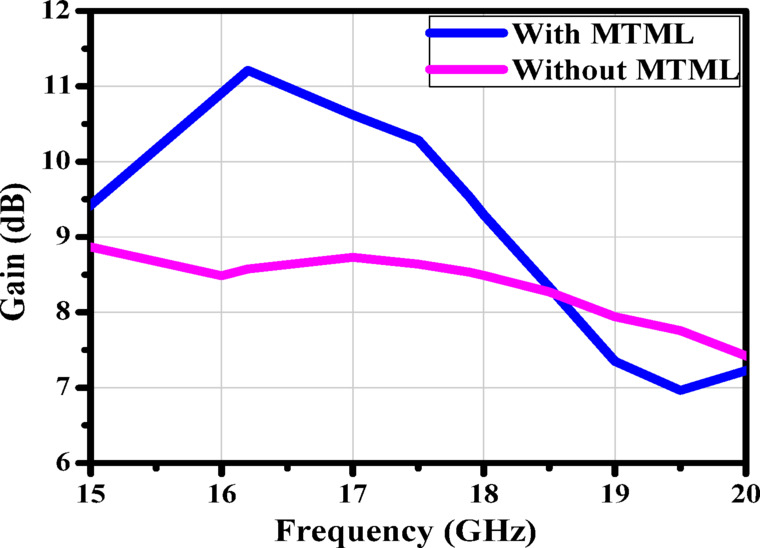
The gain values of the proposed antenna array without and with MTML.

**Fig. 17:**
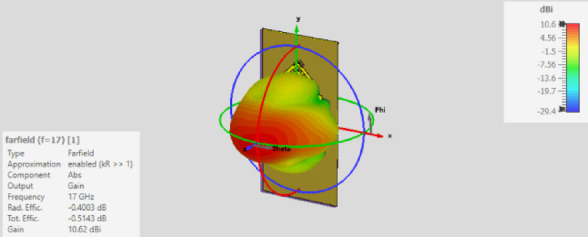
The radiation pattern of the proposed antenna array with metamaterial.

Six MTML cells are positioned vertically and rotated 45° on the x and y axes of the antenna substrate in the antenna array depicted in Fig. 14. To improve the antenna gain, these cells are oriented toward the antenna radiation. The simulated S-parameters of the suggested antenna, which covers the frequency range of 16.5–17.3 GHz, are displayed in Fig. 15. The gain value of the suggested antenna array with and without MTML cells is shown in Fig. 16. The addition of MTML cells is observed to result in a gain improvement of roughly 3 dB. The antenna array 3D radiation pattern using MTML at 17 GHz is displayed in Fig. 17.

### 4 × 1 array configurations

#### Four-element antenna array loaded without MTML

**Fig. 18:**
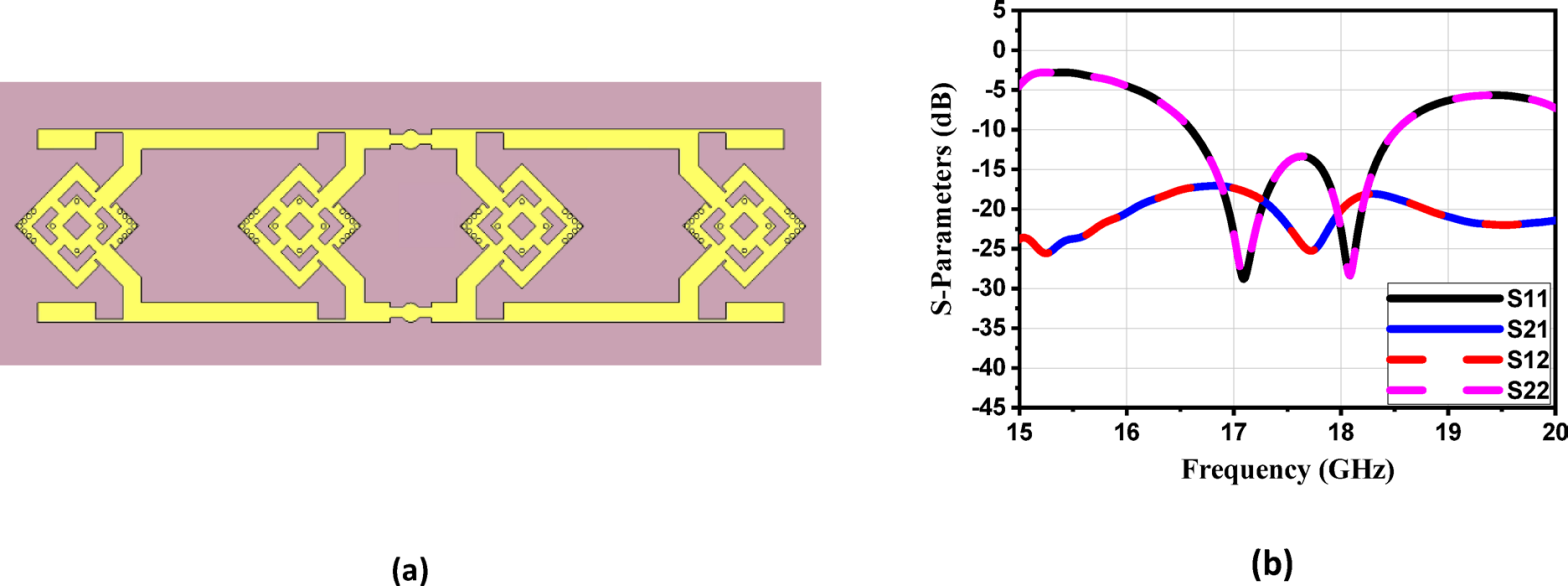
(**a**) The 4 × 1 antenna array proposed structure and (**b**) Simulated S-parameters of the proposed array.

**Fig. 19:**
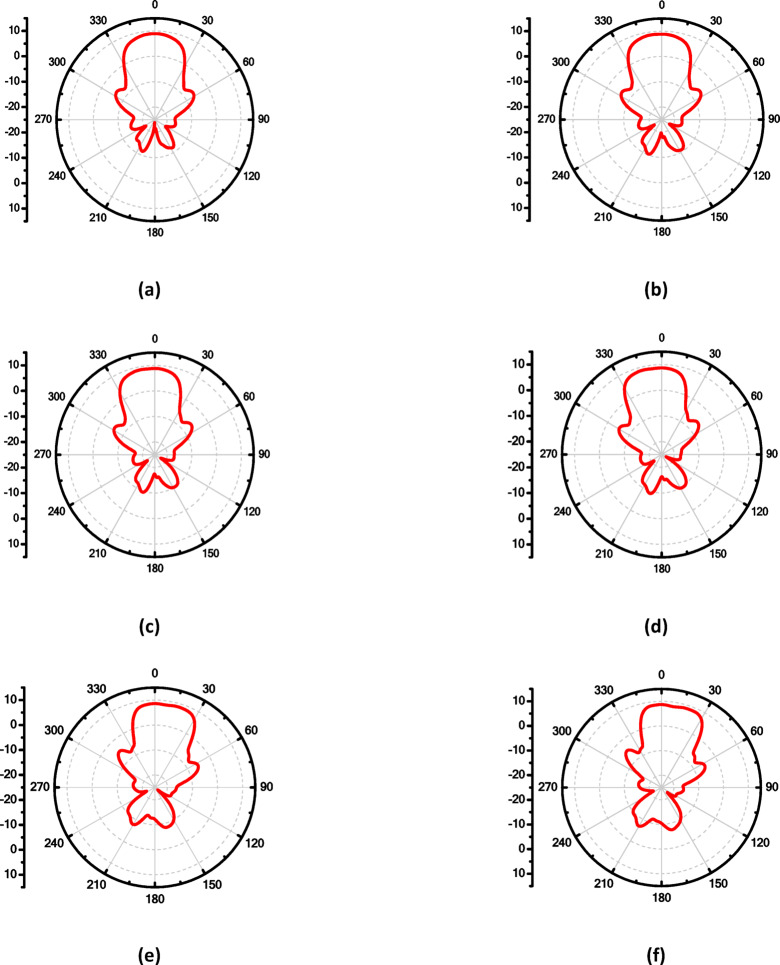
The maximum gain at different frequencies of the proposedantenna array in E-plane, (**a**) 16.6 GHz, (**b**) 16.7 GHz, (**c**) 16.8 GHz, (**d**)16.9GHz, (**e**) 17 GHz and (**f**) 17.1 GHz.

**Fig. 20:**
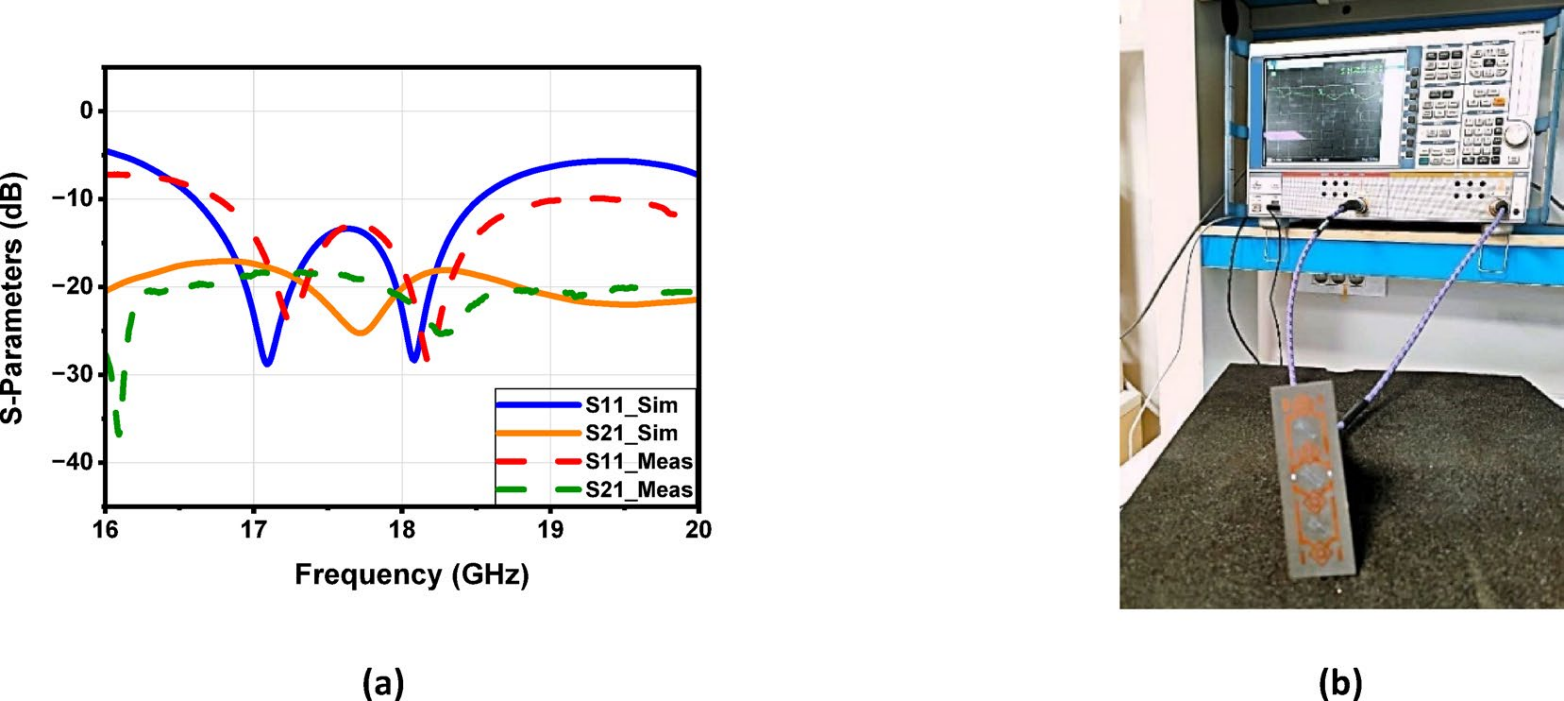
(**a**) The suggested structure’s simulated and measured S-parameters; and (**b**) the suggested antenna array’s measurement configuration.

To enhance the antenna’s gain and directivity, a 4 × 1 linear antenna array was designed, as illustrated in Fig. 18a. This array consists of four identical radiating elements. The inter-element spacing was carefully chosen between λ/2 and λ to balance mutual coupling and beam shaping performance. Additionally, two λg/2 open-circuit stubs were incorporated into the feed network to reflect the power propagating toward the ends of the feedline, thereby improving the amplitude distribution across the elements. The simulated return loss of the proposed 4 × 1 array, shown in Fig. 18b, demonstrates good impedance matching over the frequency range from 16.6 to 18.5 GHz, indicating effective broadband operation. Figure 19 illustrates the simulated farfield radiation characteristics of the proposed antenna array over the frequency range from 16.6 GHz to 17.1 GHz. Across this band, the array maintains a stable main-beam profile with gain values varying from 8.9 to 8.6 dB. The half-power beamwidth (HPBW) remains consistent, ranging approximately from 42.2° to 48.6° in elevation plane (xz-plane, φ = 0°), indicating robust beam shaping with minimal distortion over the operating frequencies. Additionally, the antenna demonstrates low (SLL), recorded between − 14.7 and − 13.3 dB, confirming effective suppression of undesired radiation and improved directional performance. These results collectively verify the array’s stable radiation behavior and suitability for operation within the targeted 16.6–17.1 GHz band. Similar radiation characteristics are observed at adjacent frequencies of 16.7 GHz, 16.8 GHz, 16.9 GHz, 17 GHz, and 17.1 GHz, as presented in Fig. 19b, c, d, e and f, respectively. Figure 20a illustrates the comparison between the simulated and measured S-parameters result of the proposed antenna. Both responses align well, with slight differences attributed to fabrication inaccuracies and measurement conditions. The antenna achieves a return loss below − 10 dB across the target frequency bands, indicating effective impedance matching. Figure 20b displays the measurement setup using a vector network analyzer VNA connected to the fabricated antenna prototype.

#### Four-element antenna array loaded with MTML

**Fig. 21:**
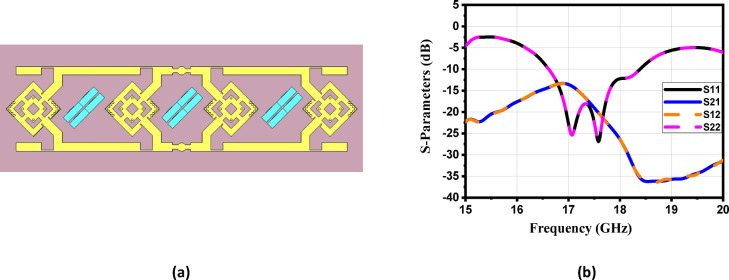
(**a**) The 4 × 1 antenna array proposed structure and (**b**) Simulated S-parameters of the proposed array.

**Fig. 22:**
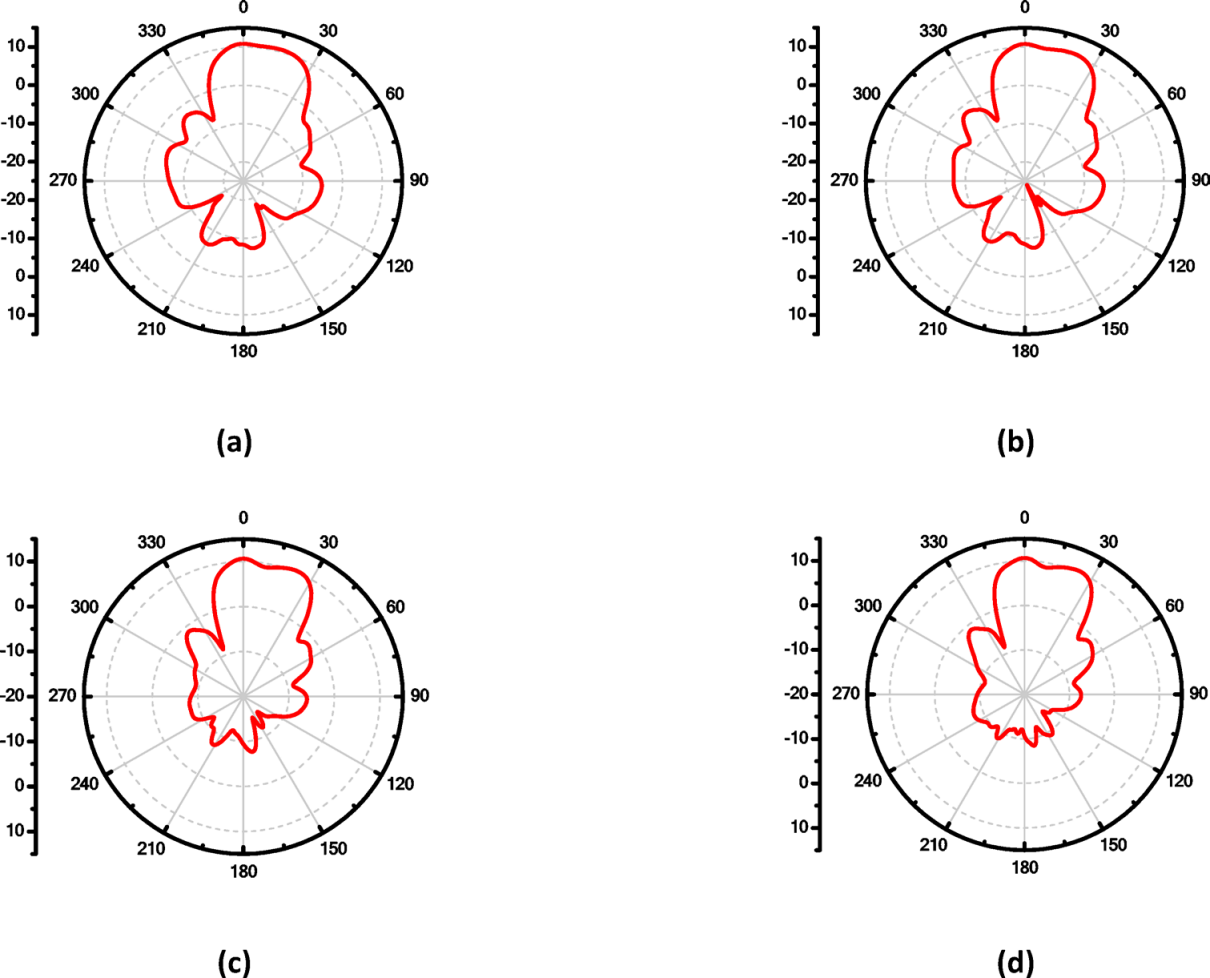
The maximum gain at different frequencies of the proposed antenna array with co-polarization in E-plane, (**a**) 16.8 GHz, (**b**) 16.9 GHz, (**c**) 17 GHz and (**d**)17.1 GHz.

For more enhancement in antenna gain and directivity, metamaterial cells are added in the 4 × 1 linear antenna array, as illustrated in Fig. 21a. This array consists of four identical radiating elements; each integrated with 12 metamaterial-inspired transmission-line (MTML) cells that are strategically oriented with a 45° rotation along both the x and y axes of the substrate. The simulated return loss of the proposed $4\times 1$ array, shown in Fig. 21b, demonstrates good impedance matching over the frequency range from 16.6 to 18.3 GHz, indicating effective broadband operation. The corresponding 2D radiation patterns in Fig. 22 show that the array achieves a peak gain of 10.6 dBi at 17 GHz. Despite the high gain performance, the antenna suffers from relatively high Side Lobe Levels (SLL)—reaching up to − 13.6 dB, along with moderate HPBWs of 40.6° in elevation plane (xz-plane, φ = 0°). Similar radiation characteristics are observed at adjacent frequencies of 16.9 GHz, 17 GHz, and 17.1 GHz, as presented in Fig. 22b, c, and d, respectively.

**Fig. 23:**
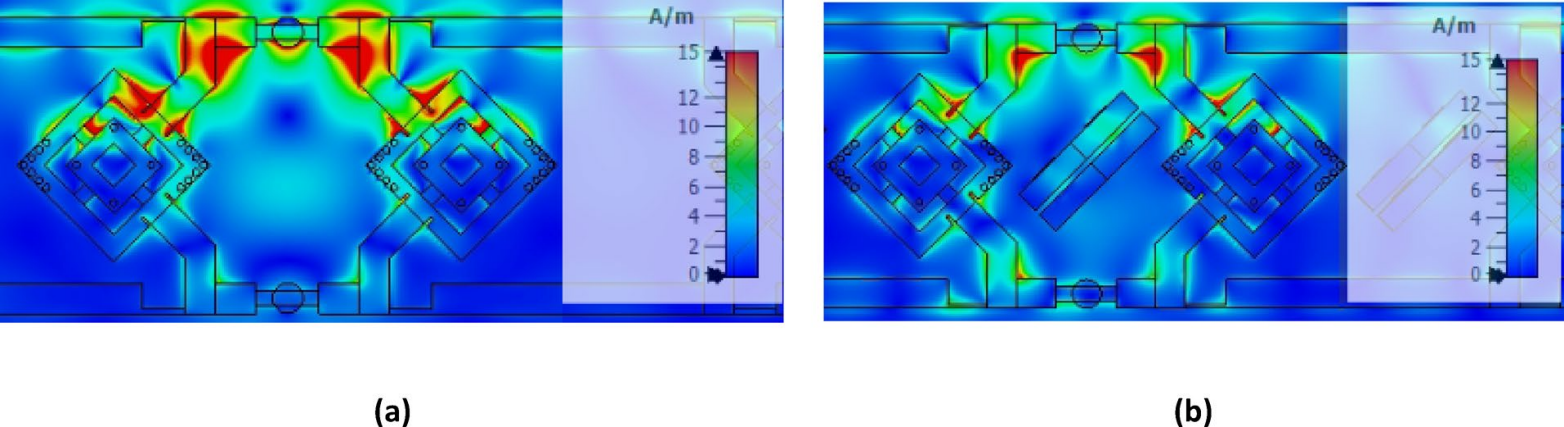
The current distribution of the proposed antenna array (**a**) Without MTML (**b**) Without MTML.

Figure 23 shows the surface current distribution of the proposed antenna without and with MTML structures. In the absence of MTML, strong surface currents are observed propagating between adjacent elements, indicating significant surface-wave coupling. When MTML unit cells are introduced, the surface currents in the inter-element region are significantly suppressed, and the fields become more localized around each radiating element. This confirms that the MTML structures effectively suppress surface-wave propagation and improve isolation, rather than acting as simple parasitic elements.

**Fig. 24:**
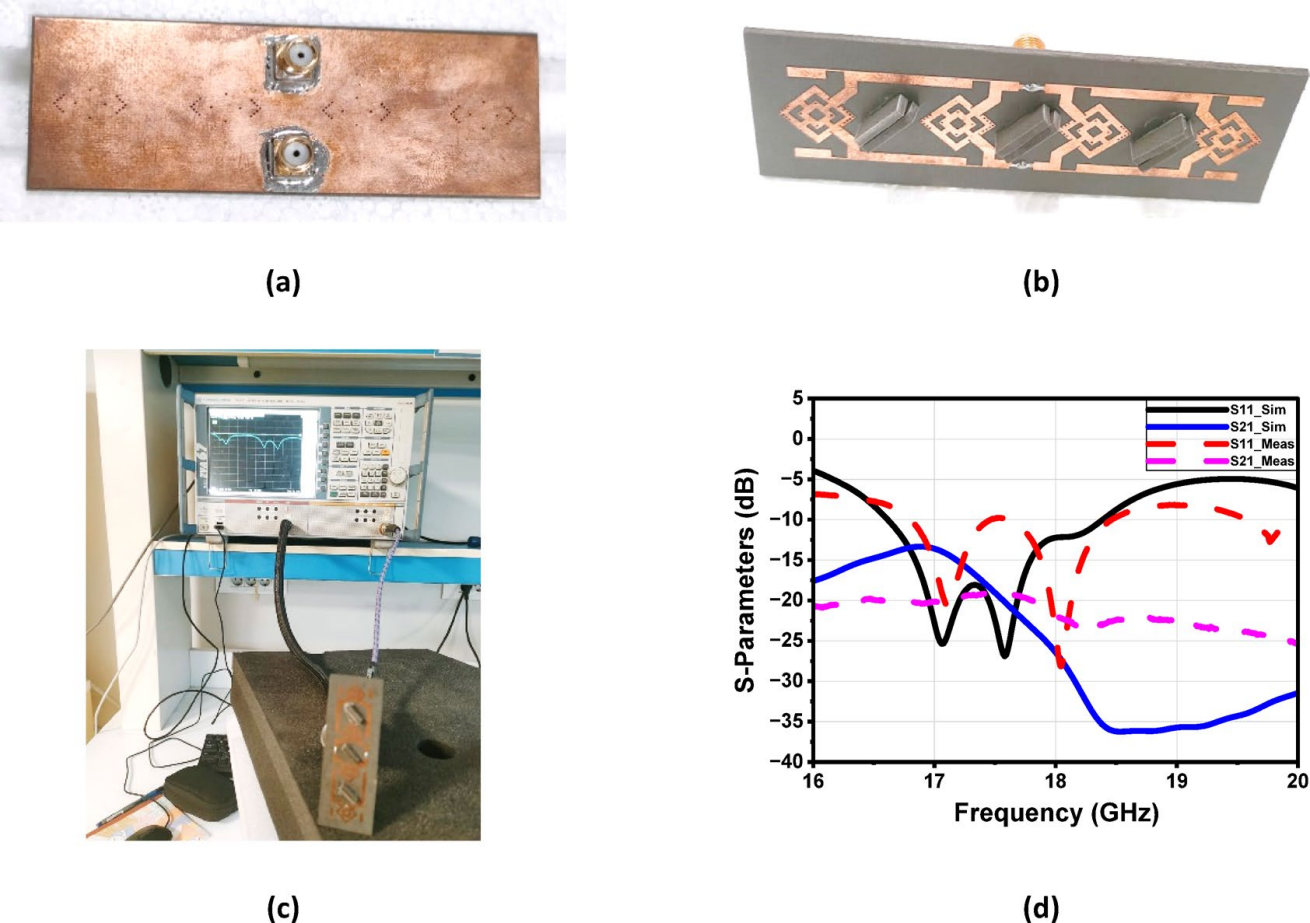
The fabrication of the proposed antenna array with MTML (**a**) Front view, (**b**) Back view, (**c**) The measurement setup and (**d**) The simulated and the measured S-parameters.

Figure 24 illustrates the fabrication and evaluation of the proposed antenna array integrated with a MTML structure. The proposed structure after the fabrication shown in Fig. 24a, b. The measurement setup, shown in Fig. 24c. The fabricated prototype was tested to validate the simulated results. Figure 24d presents a comparison between the simulated and measured S-parameters, demonstrating good agreement and confirming the antenna’s effective performance within the intended frequency band. While the MTML-based antenna array demonstrates improved gain and bandwidth, the elevated SLL remains a limitation, potentially leading to increased interference and degraded angular resolution in practical applications. To address this issue and further enhance the radiation pattern, a dielectric superstrate will be introduced above the array at λ/2, aiming to suppress sidelobe radiation, improve beam focusing, and increase overall gain and directivity.

GB-SAR sensors are subject to extreme environmental demands, including wide temperature fluctuations (from − 40 °C to over 85 °C), mechanical shock, vibration, and exposure to moisture, ice, and other harsh weather. To ensure reliable performance under these conditions, careful selection of robust materials, manufacturing processes, and packaging is critical. This includes choosing substrate materials with stable electrical and physical properties at frequencies across the operational temperature range and low moisture absorption. Furthermore, to enhance durability and maintain consistent antenna performance, the integration of protective superstrates is highly beneficial. These superstrates act as a shield, safeguarding the sensitive antenna elements from physical and environmental damage without compromising their radio frequency functionality^44^.

### Superstrate enhancement

#### One Layer superstrate four-element antenna array loaded With MTML

**Fig. 25:**
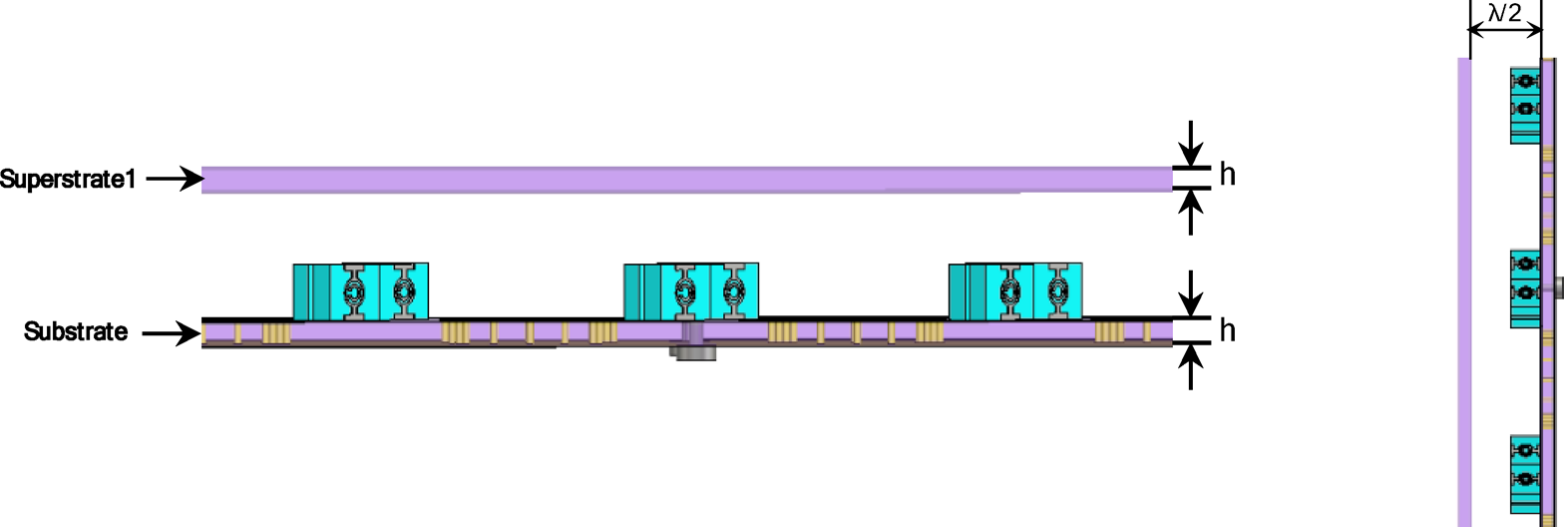
The 4 × 1 antenna array proposed structure with one layer superstrate.

To enhance the performance of the proposed 4 × 1 antenna array, a single-layer RT Duroid 5880, with a relative permittivity of 2.2, loss tangent 0.0009 and a thickness of 1.57 mm superstrate was introduced above the radiating elements, as illustrated in Fig. 25. The superstrate layer operates based on the partially reflective surface (PRS) principle, forming a Fabry–Pérot resonant cavity between the radiating antenna and the superstrate. When the spacing between the antenna and the superstrate is approximately ${\uplambda }/2$, where λ corresponds to the wavelength at the center frequency of operation. This spacing was chosen to maximize constructive interference in the forward direction and improve overall radiation characteristics. This behavior is consistent with previously reported Fabry–Pérot cavity antenna theory^45^.

**Fig. 26:**
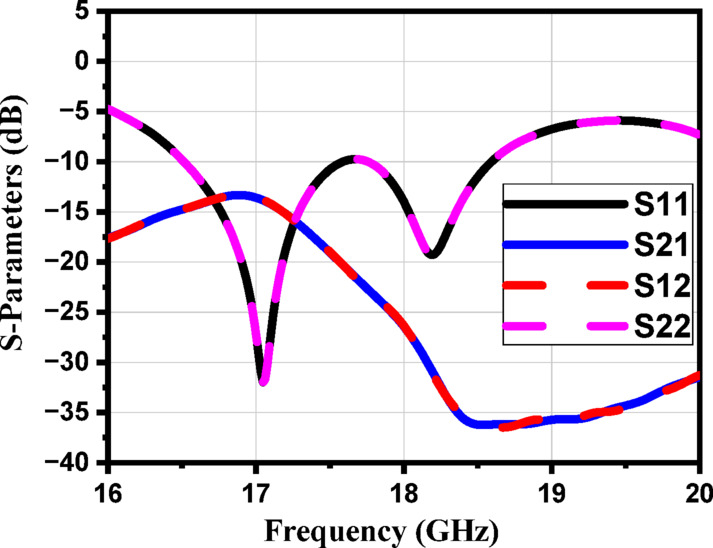
The simulated S-parameters of the proposed array with one layer superstrate.

**Fig. 27:**
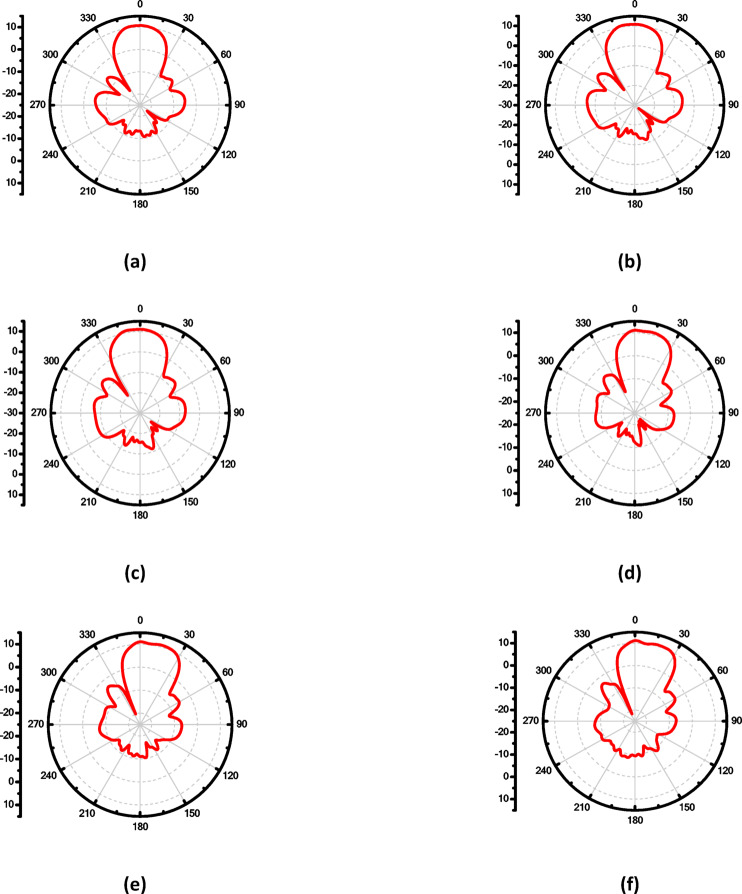
The maximum gain, SLL and HPBW with co-polarization in the elevation plane (xz-plane, φ = 0°) at different frequencies of the proposed antenna array with one layer superstrate (**a**) 16.5 GHz, (**b**) 16.6 GHz, (**c**) 16.7 GHz, (**d**) 16.8 GHz, (**e**) 16.9 GHz and (**f**) 17 GHz.

The inclusion of the superstrate led to significant improvements in the antenna’s electromagnetic performance. As shown in Fig. 26, the simulated S-parameters indicate good impedance matching and high isolation across the frequency range of interest (16.5–18.3 GHz), supporting dual-port operation with minimal mutual coupling. Furthermore, the simulated farfield radiation patterns shown in Fig. 27 at different frequencies (16.5–17 GHz) confirm that the superstrate substantially enhances the antenna’s gain, narrows the HPBW in elevation plane (xz-plane, φ = 0°), and reduces the SLL.

Without the superstrate, the antenna array exhibited a maximum gain of less than 10.6 dBi, with HPBW values exceeding 45° and SLL around − 12 dB. In contrast, with the superstrate in place, the array achieved a main lobe gain ranging from 10.8 to 11.2 dBi, peaking at 17 GHz. The HPBW was reduced to a minimum of 35.7°, indicating sharper beam formation and enhanced gain. Additionally, the SLL was significantly suppressed, reaching values as low as − 16.7 dB, thereby reducing undesired radiation and improving pattern purity.

**Fig. 28:**
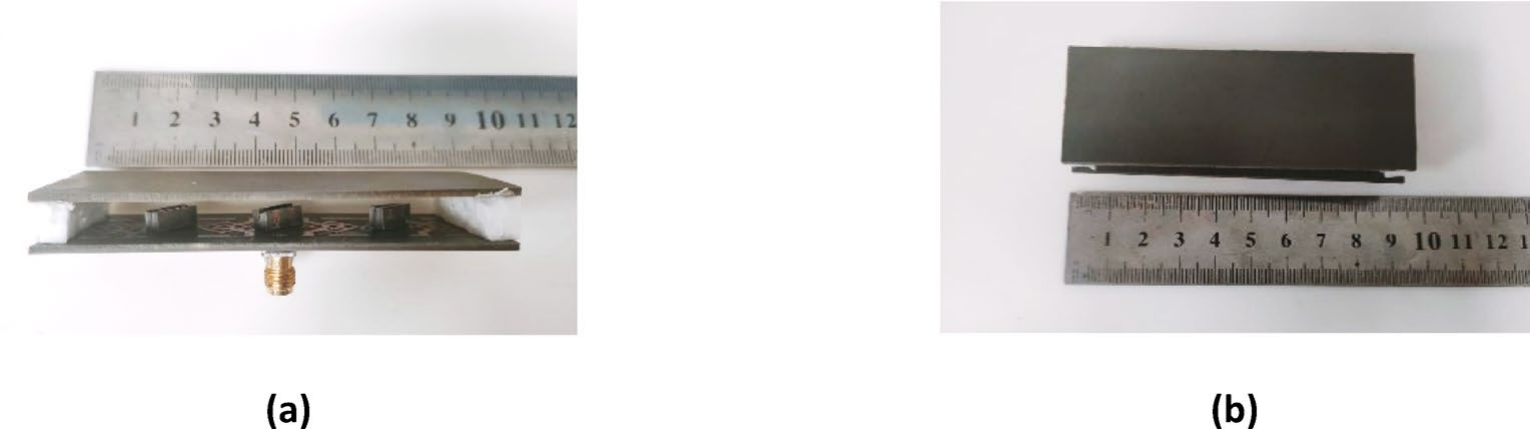
The fabrication of the proposed array with one layersuperstrate.

**Fig. 29:**
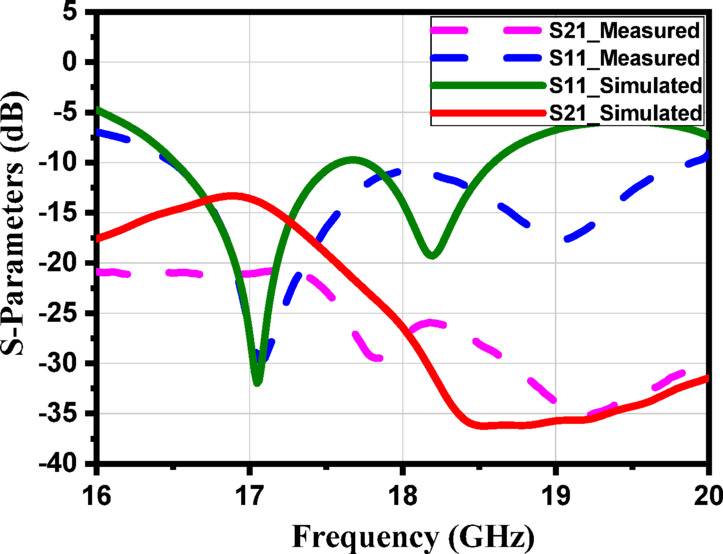
The simulated and the measured S-parameters of the proposed structure.

The fabricated prototype is shown in Fig. 28, demonstrating the physical realization of the design. The simulated and measured S-parameters are illustrated in Fig. 29, where good agreement between both results is within the desired frequency range.

**Fig. 30:**
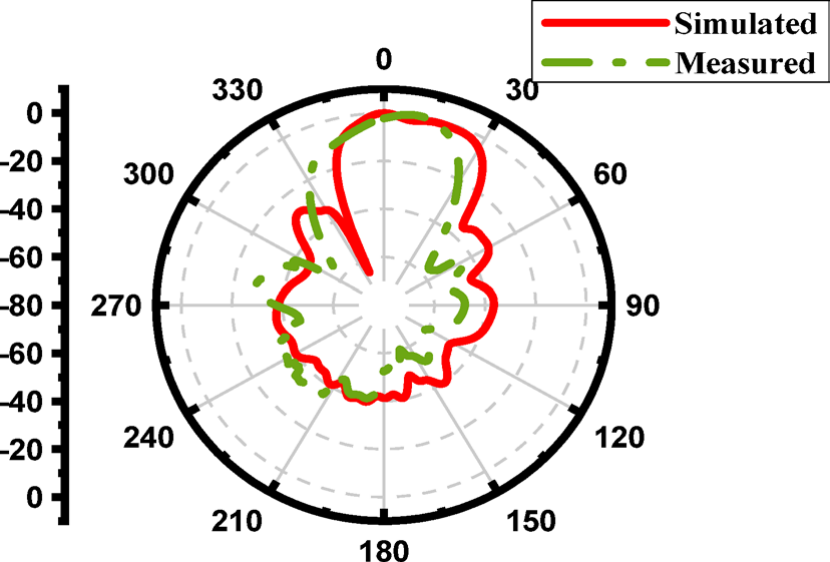
The measured and the simulated E-plane radiation pattern of the proposed antenna array with one layer superstrate at 17 GHz.

Figure 30 illustrates the simulated and measured radiation patterns of the proposed antenna array with a single-layer superstrate at 17 GHz. The close agreement between both results confirms the design’s accuracy and the superstrate’s effectiveness in improving the antenna’s directional performance. These improvements demonstrate the impact of the λ/2 spaced superstrate in enhancing the antenna array’s performance by increasing gain, narrowing the beamwidth, improving gain, and suppressing sidelobes, making the design more suitable for high-resolution and long-range sensing applications. To achieve further suppression of the SLL, an additional superstrate layer was introduced at λ/2 above the first superstrate layer, forming a multilayer structure optimized for enhanced radiation control.

#### Two layer superstrate four-element antenna array loaded With MTML

**Fig. 31:**
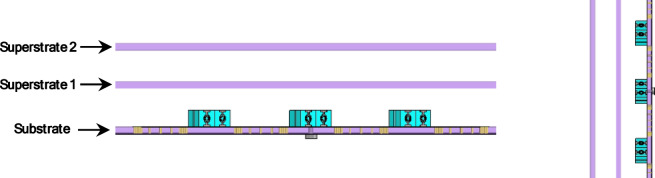
The 4 × 1 antenna array proposed structure with two-layer superstrate.

**Fig. 32:**
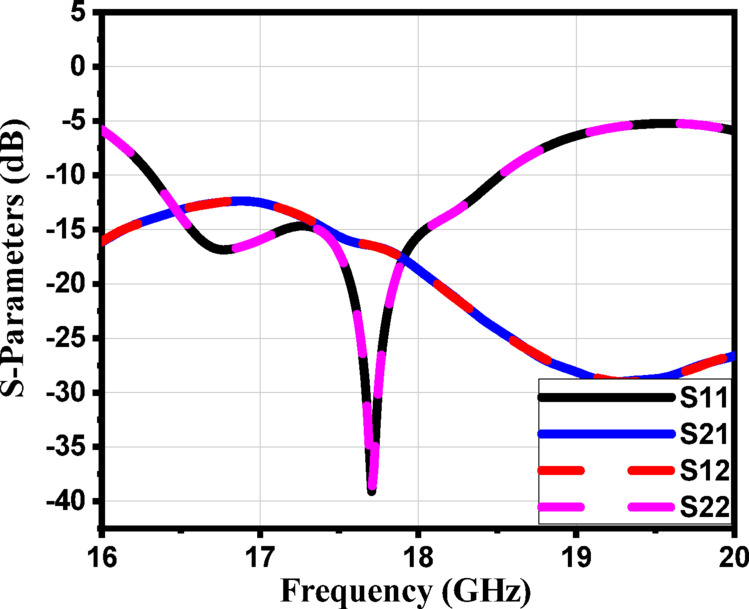
The simulated S-parameters of the proposed array with a two-layer superstrate.

The performance of the proposed 4 × 1 antenna array structure with a two-layer superstrate, shown in Fig. 31, demonstrates notable improvements in key radiation characteristics such as side lobe level (SLL) and half power beamwidth (HPBW), as verified by both S-parameters and far-field analysis. The simulated S-parameters in Fig. 32 indicate good impedance matching across a frequency band (16.3 –18.5 GHz), supporting the operational bandwidth of the antenna array.

**Fig. 33:**
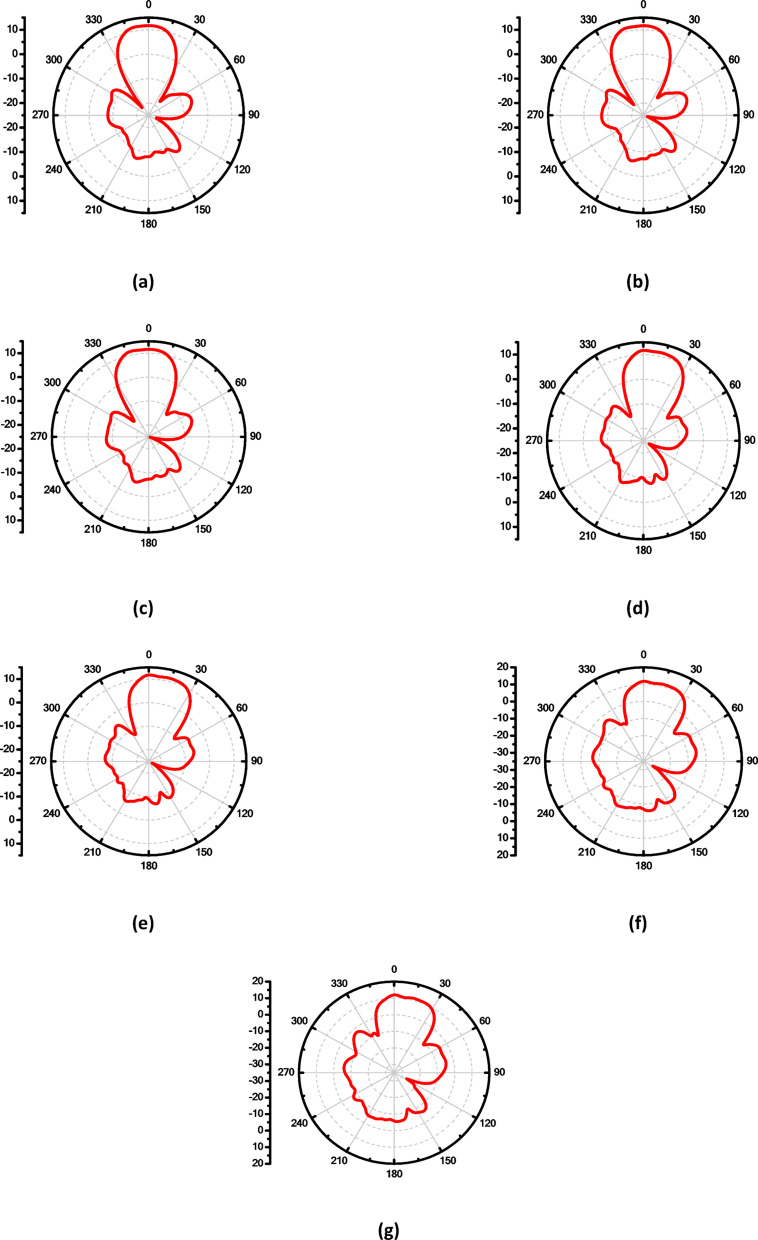
The maximum gain, SLL and HPBW with co-polarization at different frequencies of the proposed antenna array with two-layer superstrate in E-plane (**a**) 16.4 GHz, (**b**) 16.5 GHz, (**c**) 16.6 GHz, (**d**) 16.7 GHz, (**e**) 16.8 GHz, (**f**) 16.9 GHz and (**g**) 17 GHz.

The farfield radiation patterns presented in Fig. 33 (subfigures a–g) reveal that the two-layer superstrate effectively reduces SLL across multiple frequencies. Specifically, at 16.4 GHz (Fig. 33a), the SLL is − 17.6 dB with an HPBW of 36.8°, and at 16.5 GHz (Fig. 33b), the SLL is − 17.3 dB with an HPBW of 37.5°. At 16.6 GHz (Fig. 33c), the antenna achieves a SLL of − 16.9 dB and a beamwidth of 38.1°, while at 16.7 GHz (Fig. 33d), the SLL is − 17.6 dB and the HPBW is 37.9°. Furthermore, (Fig. 33e) shows the performance at 16.8 GHz, where the SLL is − 16.8 dB with an HPBW of 38.3°, and (Fig. 33f) at 16.9 GHz yields an SLL of − 15.4 dB and HPBW of 38°. Finally, at 17 GHz (Fig. 33g), the SLL is slightly higher at − 14.5 dB, with an HPBW of 38.6°. These results collectively demonstrate that the integration of the two-layer superstrate significantly enhances the radiation characteristics of the antenna array by achieving consistently low side lobe levels (below − 14.5 dB across the range) and maintaining a narrow beamwidth, which is crucial for high-gain and low-interference applications.

**Fig. 34:**
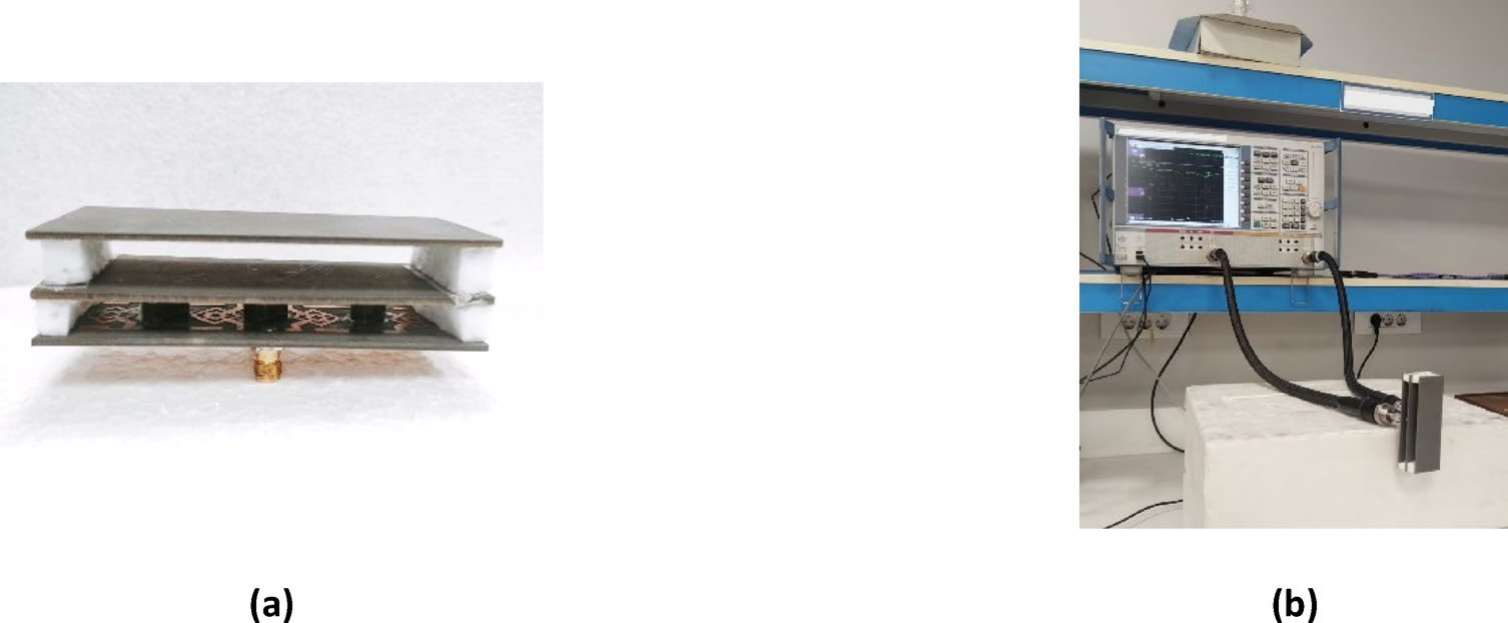
(**a**) The fabrication of the proposed array with two-layersuperstrate, and (**b**) The measurement setup of the proposed structure

Figure 34a illustrates the fabricated prototype of the proposed antenna array with the two-layer superstrate, while Fig. 34b presents the corresponding measurement setup. A good agreement between the measured and the simulated S-parameters of the proposed structure is shown in Fig. 34.

**Fig. 35:**
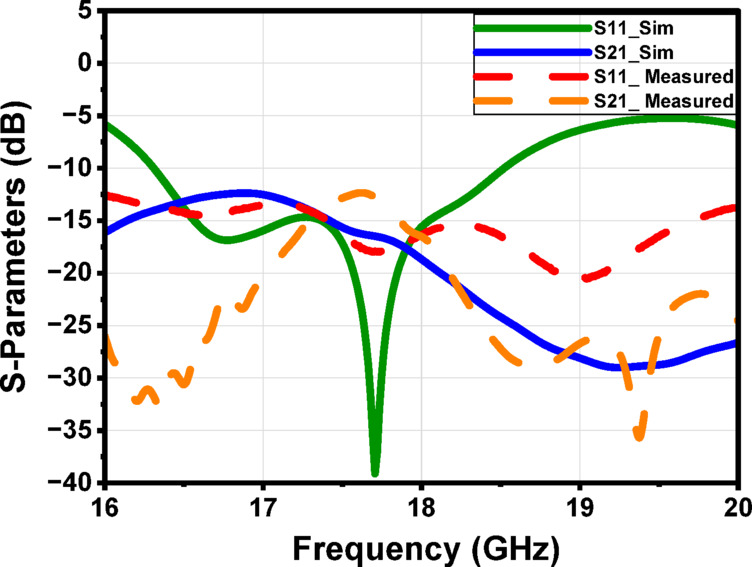
The simulated and the measured S-parameters of the proposed structure.

Figure 36 illustrates the simulated and measured radiation patterns of the proposed antenna array with two-layer superstrate at 16.8 GHz. A good agreement between both results confirms the design’s accuracy and the superstrate’s effectiveness in improving the antenna’s directional performance.

**Fig. 35:**
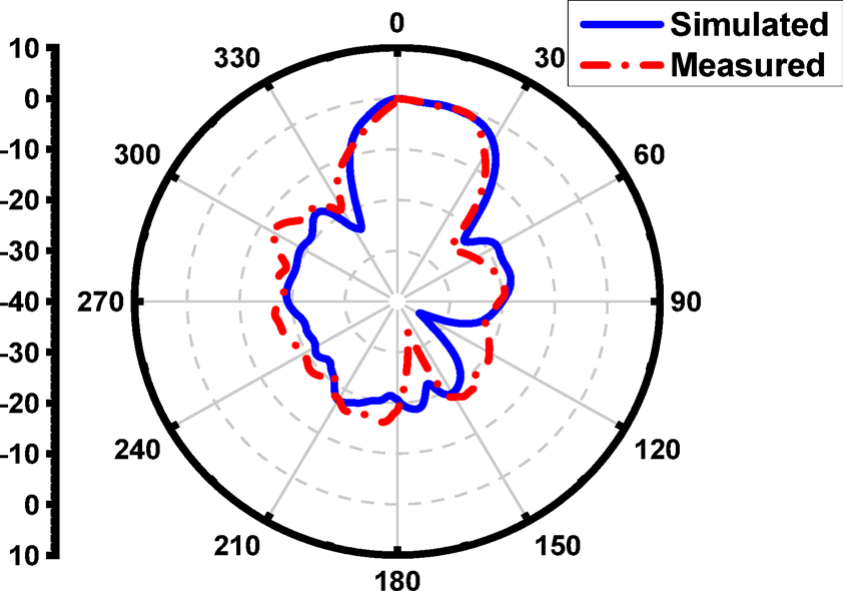
The measured and the simulated E-plane radiation pattern of the proposed antenna array with two-layer superstrate at 16.8 GHz.

**Fig. 37:**
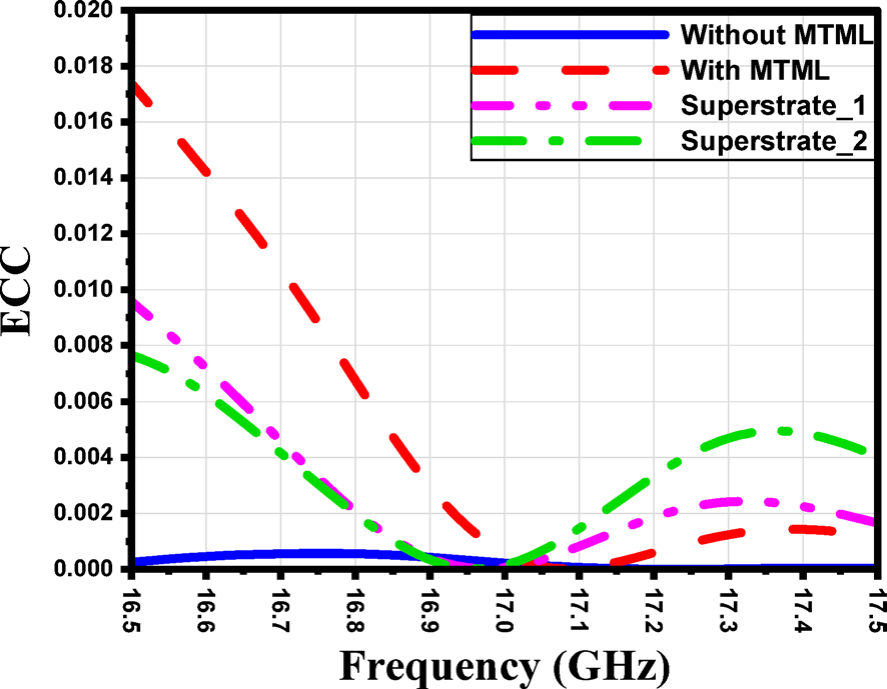
The simulated ECC of the proposed 4 × 1 antenna array without MTML, with MTML, with one-layer superstrate and with two-layer superstrate.

Figure 37 illustrates the simulated envelope correlation coefficient (ECC) of the proposed 4 × 1 antenna array without MTML, with MTML, with one-layer superstrate and with two-layer superstrate.

The ECC was calculated from the radiation patterns, which provide a more accurate evaluation of the correlation by accounting for the full 3D far-field characteristics of the antenna.

The ECC was calculated using as^46^:

9 $$\mathrm{ECC}=\frac{{\mid \iint {\overrightarrow{F}}_{1}(\theta ,\phi )\cdot {\overrightarrow{F}}_{2}^{*}(\theta ,\phi ){\hspace{0.17em}}d{\Omega }\mid }^{2}}{\left(\iint \mid {\overrightarrow{F}}_{1}(\theta ,\phi ){\mid }^{2}{\hspace{0.17em}}d{\Omega }\right)\left(\iint \mid {\overrightarrow{F}}_{2}(\theta ,\phi ){\mid }^{2}{\hspace{0.17em}}d{\Omega }\right)}$$

where ${\overrightarrow{F}}_{1}$and ${\overrightarrow{F}}_{2}$represent the far-field radiation patterns of the two ports when port (1) and port (2) are excited, respectively. where ${\overrightarrow{F}}_{1}$and ${\overrightarrow{F}}_{2}$represent the far-field radiation patterns of the two ports. θ, φ and ${\Omega }$ are solid angels. As shown in Fig. 37 the ECC remains extremely low for all configurations across the operating band. Table 1 compares the performance of four antenna array configurations across three frequencies (16.8, 16.9, and 17 GHz). The results demonstrate that the incorporation of superstrate layers significantly enhances antenna performance. Compared to the baseline array without MTML, the configuration with two superstrate layers achieves the highest gain (close to 12.0 dB), the narrowest half-power beamwidth (as low as 38.3°), and improved sidelobe suppression (down to − 16.8 dB). In addition to radiation improvements, all configurations maintain high radiation efficiency, ranging between 93.8% and 97.1% across the investigated frequencies. The proposed superstrate-loaded designs consistently exhibit efficiencies above 95% in most cases, confirming that the performance enhancement does not introduce significant loss. Furthermore, the ECC remains extremely low for all configurations, with values below 0.006 across the operating band and reaching as low as 0.00006 at 17 GHz. These very small ECC values indicate excellent port isolation and outstanding diversity performance. Overall, the improvements are consistent across all tested frequencies, demonstrating that the use of multiple superstrate layers enhances gain, focuses the radiation pattern, suppresses sidelobes, while simultaneously preserving high efficiency and very low correlation, thereby offering a robust solution for high-frequency antenna array applications.

**Table 1:** Comparison between the proposed antenna array configurations at different frequencies.

Antenna type	Frequency (GHz)	Gain (dB)	HPBW (°)	SLL (dB)	Mutual coupling (dB)	Radiation Efficiency (%) (Sim.)	ECC
1- Array without MTML	16.8	8.76	44.1	– 14.4	<– 16.9	96.8	0.0005
2- Array with MTML		10.8	40.6	– 13.6	<– 13.4	97.1	0.006
3- One-layer superstrate		11.1	38.7	– 15.3	<– 13.4	96.9	0.002
4- Two-Layer superstrates		11.8	38.3	– 16.8	<– 12.5	96.71	0.0019
1- Array without MTML	16.9	8.68	45.2	– 13.9	<– 17.1	95.8	0.0004
2- Array with MTML		10.7	42.4	– 12.6	<– 13.3	94	0.002
3- One-Layer superstrate		11.1	40.6	– 14.6	<– 13.3	94.7	0.0004
4- Two-Layer superstrates		12	38.9	– 15.5	<– 12.4	95.1	0.0003
1- Array without MTML	17	8.6	46.2	– 13.5	<– 17.4	95	0.0002
2- Array with MTML		10.6	43.8	– 11.9	<– 13.5	93.8	0.0005
3- One-Layer superstrate		11.2	41.7	– 14.4	<– 13.3	94.9	0.00006
4- Two-Layer superstrates		12	38.5	– 14.9	<– 12.5	95.4	0.00015

Table 2 compares the proposed antenna design with existing ones. According to Table 2, a competitive antenna design that achieves a balance of performance through structural simplicity. While many referenced works use multi-layer structures (3–7 layers) and large arrays to reach higher gains, our single-layer, 4 × 1 linear array design attains a gain of 11.08 dB across 16.3–18.5 GHz with a compact 90 × 30 mm footprint and a practical HPBW of 38.3°. The key contribution is demonstrating that excellent performance in gain, beamwidth, and sidelobe level (– 16.8 dB) can be realized with a minimal, low-complexity architecture, offering a cost-effective and easily integrable solution without the drawbacks of multi-layer fabrication.

**Table 2:** Comparison between the proposed result and the previous work.

References	BW (GHz)	Dimensions (mm)	Gain (dB)	No. of layers (single element)	Array	HPBW (°)	SLL (dB)	Feeding mechanism	Design method	Mutual Coupling (dB)	Polarization	Complexity
^47^	14–16	300 × 300	5.4–7.4	3 layers	8 × 8	Not reported	– 10	Aperture-coupled stacked patch	Shared Aperture	-33	DBDP	High
^48^	14.6–15.6	96 × 96	18.1	3 layers	4 × 4	Not reported	– 15	Proximity coupled	Shared Aperture	<– 30	Dual	Medium
^49^	9.28–12.96	14.55 × 14.55	6.63	6 layers	1	Not reported	Not reported	Four-Port Shared-Aperture	L-probe	<– 12	Dual	High
^50^	14–18	55 × 55	17.13	7 layers	4 × 4	55	– 11	ADS + DGS	Cross-shaped slot	<– 35	Dual	High
^51^	11.1–12.4	57.9${{\uplambda }}_{\mathrm{o}}^{2}$ × 62.35 ${{\uplambda }}_{\mathrm{o}}^{2}$	21	3 layers	22 × 22	30	– 20.8	3-bit Phase Compensation Transmitarray	SIW 2 × 2 array feed	Not reported	Dual	Medium
^52^	17.02–17.4	110 × 149	25	1 layer	2 × 8	8	– 16	Offset Cylindrical Reflector	Micro-strip patch array	Not reported	linear	High
This paper	16.3–18.5	90 × 30	12	1 layer	4 × 1	38.3	– 16.8	Microstrip transmission line	Micro-strip patch array	<– 12.5	Dual	Low

## Conclusion

This work presents a compact and high-efficiency 4 × 1 dual-polarized square-ring patch antenna array designed for Ku-band GB-SAR applications (16.8–17 GHz). The proposed same-level dual-port configuration successfully achieves dual polarization within a single radiating layer while maintaining good impedance matching and radiation symmetry, which is typically challenging in compact dual-port structures.

Comprehensive analysis demonstrates that the integration of metamaterial unit cells (MTMLs) between adjacent elements effectively suppresses surface waves and reduces inter-element coupling. Across the investigated frequency range, mutual coupling is maintained below − 12.5 dB, while radiation efficiency remains high, varying between 93.8% and 97.1%. Additionally, the Envelope Correlation Coefficient (ECC) remains extremely low (below 0.006 for all configurations), confirming excellent diversity and polarization isolation performance suitable for MIMO radar systems. The incorporation of dielectric superstrates further enhances radiation characteristics. The two-layer superstrate configuration achieves the best overall performance, providing a peak gain of 12 dBi, a narrowed half-power beamwidth down to 38.3°, and sidelobe level suppression reaching − 16.8 dB. These improvements indicate enhanced beam focusing capability and improved angular resolution, which are critical for high-accuracy radar sensing. Beyond electromagnetic improvements, the superstrate layers also provide mechanical robustness and environmental protection, acting as a shielding structure that safeguards the antenna against temperature variations, vibration, and moisture exposure commonly encountered in automotive environments. Overall, the proposed antenna system demonstrates a balanced combination of compact size, high radiation efficiency, low mutual coupling, excellent diversity performance, and enhanced beam control. The presented design validates the feasibility of integrating metamaterial loading and protective superstrate techniques in compact dual-polarized arrays, making it a strong candidate for advanced Ku-band automotive radar and sensing applications.

## Data Availability

No/Not applicable. This manuscript does not report data generation or analysis.
